# VAV2 exists in extrachromosomal circular DNA and contributes Enzalutamide resistance of prostate cancer via stabilization of AR/ARv7

**DOI:** 10.7150/ijbs.109271

**Published:** 2025-04-09

**Authors:** Qinghua Wang, Xin'an Wang, Hanchu Ye, Yicong Yao, Haopeng Li, Xin Qin, Yan Zhao, Wei Jiang, Mingming Xu, Tong Zi, Xilei Li, Xi Chen, Juan Zhou, Gang Wu

**Affiliations:** 1 Department of Urology, Tongji Hospital, School of Medicine, Tongji University, Shanghai 200065, China.; 2 Department of ICU, Tongji Hospital, School of Medicine, Tongji University, Shanghai, 200065, China.

**Keywords:** eccDNAs, VAV2, prostate cancer, AR/ARv7, USP48, Enzalutamide resistance

## Abstract

Extrachromosomal circular DNAs (eccDNAs) are circular, double-stranded DNA molecules ubiquitously present across various organisms, playing a critical role in tumorigenesis and tumor progression. However, their precise contribution to prostate cancer (PCa) remains incompletely understood. To elucidate the function of eccDNAs in PCa, eccDNA sequencing and annotation were performed in PCa tissues and cell lines using Circle-seq. Amplified genes on eccDNAs were identified by cross-referencing annotated eccDNA-associated genes with those overexpressed in PCa based on TCGA data. Furthermore, eccDNA profiles were compared between Enzalutamide-sensitive and -resistant cell lines to investigate their role in resistance mechanisms. Notably, VAV2 was detected on both linear and circular DNA, as confirmed by PCR and Sanger sequencing. Functional analyses demonstrated that VAV2 overexpression promotes PCa proliferation and metastasis by activating the PAK1/AKT signaling pathway through PAK1 phosphorylation. Additionally, VAV2 contributes to Enzalutamide resistance by enhancing AR/ARv7 protein stability via reduced ubiquitination, mediated through the recruitment of the deubiquitinating enzyme USP48. These findings establish VAV2, identified through eccDNA sequencing, as a potential oncogene and a promising biomarker for PCa diagnosis and prognosis.

## Background

Prostate cancer (PCa) has become one of the most prevalent malignancies of the male genitourinary system. According to the 2022 GLOBOCAN data, PCa ranks second in incidence and fifth in cancer-related mortality among men worldwide^1^. In the United States, it is now the most frequently diagnosed malignancy in men and the second leading cause of cancer-related death^2^. In China, PCa incidence has surged with an annual growth rate of approximately 4.7%^3^. Although the overall incidence remains lower than in Western countries, 20% of Chinese patients are diagnosed at an advanced metastatic stage^4^. Metastatic lesions, predominantly in bone and lymph nodes, are associated with poor prognosis and substantially increased mortality risk^5^. Currently, no curative treatment exists for metastatic PCa, and the 5-year survival rate remains below 40%^6^. Elucidating the mechanisms of advanced PCa remains a critical challenge with significant clinical implications.

Extrachromosomal circular DNAs (eccDNAs) are circular, double-stranded DNA molecules widely present across various organisms, first identified by Alix Bassel and Yasuo Hotta in 1965^7^. Despite their ubiquity, the mechanisms underlying eccDNA formation remain speculative, with no universally accepted model. Proposed mechanisms include double-strand break repair, chromosome fragmentation, and the breakage-fusion-bridge cycle^8^. EccDNAs exhibit diverse characteristics based on their origin, size, sequence, and structural attributes, encompassing mitochondrial circular DNA, episomes, small polydisperse circular DNA, double-minute chromosomes, telomeric circles, extrachromosomal DNA, and microDNA^9^, ^10^. Recent studies indicate that eccDNAs are commonly detected in healthy human tissues and blood, with potential roles in aging and other physiological processes^8^, ^11^. Notably, elevated eccDNA levels have been observed in tumor tissues, suggesting a role in tumor progression and heterogeneity^12^.

VAV2, the second member of the VAV guanine nucleotide exchange factor family, alongside VAV1 and VAV3, serves as a critical regulator of cellular processes by functioning as a molecular switch for Rho GTPases^13^, ^14^. Dysregulated Rho GTPase signaling has been implicated in multiple malignancies due to its broad regulatory functions^15^. As specific GDP/GTP nucleotide exchange factors, VAV proteins are tightly regulated through tyrosine phosphorylation^16^. Emerging evidence suggests that aberrant VAV2 expression contributes to tumor progression across various cancer types^17^-^19^.

This study initially examined the distinct expression patterns of eccDNAs in PCa, verifying the presence of each eccDNA within the corresponding chromosomal sequence. Analysis revealed that all chromosomes generate substantial amounts of eccDNAs, with ecc-VAV2 expression significantly upregulated in both PCa tissues and cell lines. Notably, Enzalutamide-resistant PCa cells exhibited increased eccDNA levels, accompanied by elevated ecc-VAV2 expression compared to Enzalutamide-sensitive counterparts. Functional assays demonstrated that elevated VAV2 expression enhances PCa cell proliferation and metastasis *via* activation of the PAK1/AKT signaling pathway. Additionally, VAV2 contributes to Enzalutamide resistance by stabilizing AR/ARv7 through USP48-mediated inhibition of ubiquitination. Identifying genes highly expressed in eccDNA form and elucidating their functional mechanisms provide valuable insights for developing novel therapeutic strategies for PCa.

## Methods

### Patient samples and cell culture

All PCa tissues and adjacent normal tissues were collected from Tongji Hospital of Tongji University between 2020 and 2024. Specimens were either immediately fixed in 4% paraformaldehyde for immunohistochemistry (IHC) or flash-frozen in liquid nitrogen for subsequent Western blot analysis. Informed consent was obtained from all patients. The RWPE-1, LNCaP, C4-2, and 22RV1 cell lines were obtained from the Cell Bank of the Chinese Academy of Sciences (Shanghai, China). To generate the Enzalutamide-resistant C4-2 cell line (C4-2R), parental C4-2 cells were initially exposed to 10 μM Enzalutamide (Abmole, China) for 30 days, followed by stepwise escalation to 20 μM and subsequently 40 μM, with each concentration maintained for 30 days. To preserve resistance, C4-2R cells were continuously cultured in a medium containing 10 μM Enzalutamide. All other cell lines were maintained in a complete medium and incubated at 37°C in a 5% CO_2_ atmosphere.

### EccDNAs enrichment and analysis

The eccDNA enrichment and analysis process involved the following steps: Cells were lysed on ice to extract high-molecular-weight (HMW) DNA, followed by exonuclease treatment to eliminate linear DNA. Upon complete removal of linear DNA, the enriched eccDNA fraction was used as a template for amplification with phi29 polymerase. The amplified DNA underwent sonication using a Bioruptor system to generate a fragmented DNA library, which was purified with magnetic beads. The size distribution of the resulting fragments was analyzed before employing Circle-MAP for eccDNA identification, gene annotation, differential eccDNA analysis, and functional enrichment of annotated genes.

### Quantitative Real-time PCR

Total RNA from PCa cells was extracted using the FastPure Cell/Tissue Total RNA Isolation Kit (Vazyme, China). Complementary DNA (cDNA) was synthesized with the ABScript Neo RT Master Mix with gDNA Remover (Abclonal, China) following the manufacturer's protocol. Quantitative PCR (qPCR) was performed using BrightCycle Universal SYBR Green qPCR Mix with UDG (Abclonal, China), with primers synthesized by Sangon Biotech (China).

### Agarose gel electrophoresis

For gel electrophoresis, a 1.5% agarose gel was prepared by dissolving agarose in TAE buffer and heating it in a microwave for 3 minutes. SafeRed dye (Abclonal, China) was then added to the solution, which was poured into a mold and allowed to solidify. DNA samples, pre-mixed with DNA loading buffer (Abclonal, China), were loaded onto the gel and subjected to electrophoresis, followed by visualization using a gel imaging system.

### Western blot

Proteins were extracted from cell lysates and tissue homogenates, with nuclear and cytoplasmic fractions isolated using a Beyotime extraction kit (China). Denatured proteins were separated via 10% SDS-PAGE and subsequently transferred onto a PVDF membrane using a Rapid Transfer Buffer (Beyotime, China) at 200 mA. To minimize nonspecific antibody binding, membranes were blocked before overnight incubation with primary antibodies specific to the target proteins, diluted in a blocking solution. Following three washes with TBST, membranes were incubated with an HRP-conjugated secondary antibody for 1 hour at room temperature. Protein bands were then visualized using an enhanced chemiluminescence substrate (Beyotime, China) and imaged with a gel documentation system. Densitometric analysis was performed using ImageJ software.

### Cell transfection assay

For VAV2 overexpression and knockdown, the oe-VAV2 plasmid (Youze, China) or Si-RNA (Youze, China) was constructed. When cells reached 50% confluency, transfection was carried out using Lipofectamine 2000 (Invitrogen, USA). qRT-PCR and Western blotting were performed 48 or 72 hours post-transfection to confirm transfection efficiency. Stable knockdown or overexpression cell lines were established for *in vivo* experiments.

### Dual-luciferase reporter assay

To assess VAV2 promoter activity, various promoter fragments were cloned into the pGL3.0-basic vector (Promega, USA). The resulting pGL3-derived constructs were co-transfected with the pRL-TK Renilla plasmid using Lipofectamine 2000 (Invitrogen, USA) according to the manufacturer's protocol. After 36 hours, dual-luciferase activity was quantified using the Dual-Luciferase Reporter Assay System (Promega, USA).

### Chromatin immunoprecipitation assay (ChIP)

ChIP was performed using the BeyoChIP™ ChIP Assay Kit (Beyotime, China) following the manufacturer's instructions. Immunoprecipitated DNA was amplified using VAV2 promoter-specific primers. PCR products were resolved on a 1.5% agarose gel containing ethidium bromide in 1 × TAE buffer. Electrophoresis was conducted at 100 V for 45 minutes, and DNA bands were visualized under UV illumination using a Gel Documentation System (Bio-Rad, USA).

### Fluorescence in Situ Hybridization assay (FISH)

The Cy3-labeled ecc-VAV2 probe and FISH assay were designed by Ribobio (China) and performed according to the manufacturer's instructions. Briefly, PCa cells were fixed in 4% paraformaldehyde and permeabilized with 0.5% Triton X-100 at 4°C. After PBS washes, cells were incubated in a pre-hybridization buffer before hybridization with the ecc-VAV2 probe at 37°C overnight. Following hybridization, sequential washes with 4 × SSC, 2 × SSC, and 1 × SSC were performed at room temperature. Nuclei were counterstained with DAPI, and fluorescence signals were captured using a confocal microscope.

### CCK-8 assay

Cell viability was assessed *via* the CCK-8 assay. Approximately 5,000 cells were seeded into 96-well plates and evenly distributed by gentle shaking. The next day, the culture medium was replaced with fresh medium containing 10% CCK-8 reagent (Beyotime, China), followed by a 2-hour incubation at 37°C. Optical density (OD) values were measured using a microplate reader.

### Colony formation assay

For colony formation assays, PCa cells (800 cells/well) were plated in 12-well plates and cultured for approximately 2 weeks, with a medium refreshed every 4 days. Once colonies had formed, cells were fixed with 4% paraformaldehyde for 15 minutes and stained with crystal violet for 30 minutes.

### EdU assay

EdU incorporation assays were conducted using the BeyoClick™ EdU Cell Proliferation Kit with Alexa Fluor 555 (Beyotime, China). Cells were seeded into 96-well plates at an appropriate density and cultured overnight for attachment. The percentage of proliferating cells was determined by calculating the ratio of EdU-positive cells to Hoechst 33342-stained nuclei.

### Transwell assay

For Transwell invasion assays, cells were seeded into the upper chamber of a Matrigel-coated Transwell insert (Corning, USA), while the lower chamber contained medium supplemented with 20% FBS to establish a chemoattractant gradient. After 48 hours of incubation, non-invaded cells on the upper membrane surface were gently removed with a cotton swab. Invaded cells on the lower surface were fixed with 4% paraformaldehyde for 20 minutes, stained with 0.1% crystal violet, and air-dried. The number of invaded cells was quantified under a microscope. Migration assays followed the same procedure, except that Matrigel coating was omitted to assess migratory capacity.

### Co-IP assay

Protein-protein interactions were analyzed using the Beyotime Immunoprecipitation Kit (China) following the manufacturer's protocol. Immunoprecipitated samples were subjected to Western blot analysis, with special secondary antibodies (Abmart, China) employed to avoid interference from IgG light and heavy chains.

### Immunofluorescence assay (IF)

PCa cells were uniformly seeded onto slides and incubated for 24 hours. Cells were then fixed with 4% paraformaldehyde and permeabilized with 0.5% Triton X-100 (Beyotime, China). To minimize non-specific antibody binding, cells were blocked with 3% BSA for 1 hour, followed by overnight incubation with the primary antibody. The next day, a fluorescent secondary antibody (Abclonal, China) was applied in a light-protected environment. Finally, an anti-fade mounting medium containing DAPI (Solarbio, China) was added, and fluorescence images were captured using a fluorescence microscope.

### Hematoxylin and Eosin (HE) staining

For HE staining, paraffin-embedded tissue sections were deparaffinized in xylene and rehydrated through a graded ethanol series to water. Hematoxylin was applied to stain the nuclei blue, followed by washing to remove excess stain. Eosin was then used to stain the cytoplasm pink. Tissue sections were dehydrated through increasing ethanol concentrations, cleared with xylene, and imaged.

### Immunohistochemistry assay (IHC)

Experimental procedures for IHC followed a previously described protocol^20^.

### Xenograft assay

Male BALB/c nude mice (4 weeks old) were obtained from Shanghai Shengchang Co., Ltd (Shanghai, China) and randomly assigned to the sh-NC and sh-VAV2 groups or the oe-NC and oe-VAV2 groups. After acclimation under specific pathogen-free (SPF) conditions for 3 days, all mice proceeded to the experimental phase. For tumorigenicity assays, 2 × 10^6^ 22RV1 or C4-2 cells suspended in 100 μL of RPMI-1640 medium were injected into the forelimb axilla. Tumor growth was monitored for 4 weeks. To investigate the role of VAV2 in Enzalutamide-resistant PCa, mice underwent surgical castration before randomization into DMSO or Enzalutamide treatment groups (10 mg/kg, orally). After 4 weeks, tumors were excised for weight measurement and IHC analysis. All animal experiments were approved by the Animal Care Committee of Tongji Hospital, Tongji University (No. 2024-DW-(085)).

### Statistical analysis

Circle-seq data analysis followed these steps: Raw data quality was assessed using FastQC. Reads were then aligned to the reference genome using BWA. SAMtools was employed to convert the alignment files into the appropriate format for Circle-MAP, which was subsequently used for eccDNA detection and gene annotation. Statistical analyses were performed using SPSS Statistics 26 and GraphPad Prism 10, with all data presented as mean ± SD. Group comparisons were conducted using the *t*-test or one-way ANOVA, while the χ^2^ test was applied to evaluate associations between protein expression and clinical characteristics. Statistical significance was set at *P < 0.05, with "ns" indicating non-significance.

## Results

### EccDNAs detection and analysis in PCa tissues

**Figure 1:**
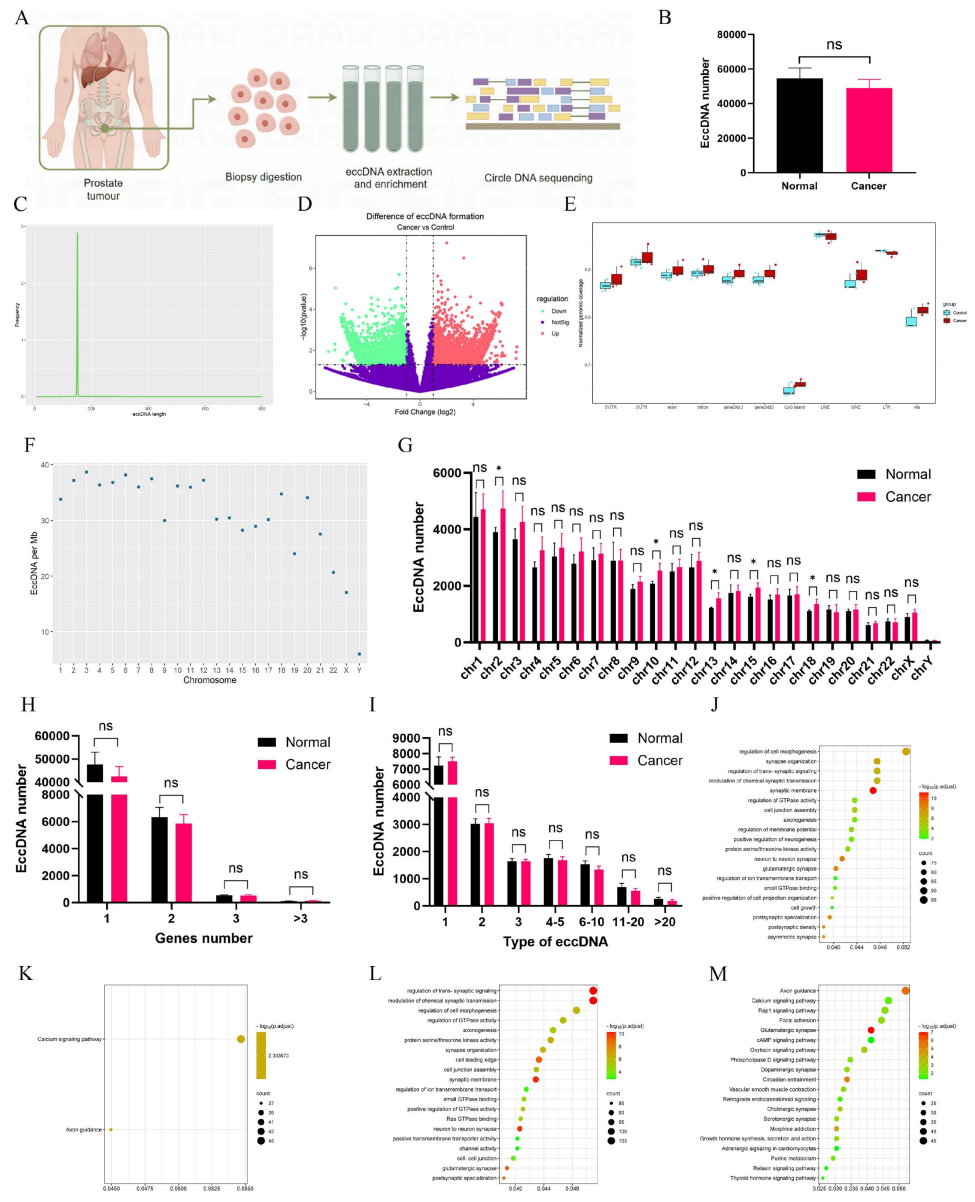
** Detection and analysis of eccDNAs in clinical tissues. A** Circle-seq was utilized to acquire sequence information on eccDNAs of PCa tissues and schematic diagram drawn by figdraw. **B** The number of eccDNAs in PCa and normal tissues. **C** The distribution of eccDNAs in tissues. **D** A volcano plot was constructed. **E** The normalized genomic coverage of eccDNAs across corresponding genomic elements. **F** The number of eccDNAs per Mb chromosome. **G** The number of eccDNAs derived from each chromosome. **H** Number of eccDNAs derived from different genes. **I** Types of eccDNAs included different types of genes. **J-M** GO and KEGG enrichment analyses on up-regulated and down-regulated eccDNA-related differential genes. *P < 0.05, "ns"is not significant.

Circle-seq was employed to obtain sequence information on eccDNAs (Fig. 1A). The average number of eccDNAs in adjacent tissues exceeded 54,000; however, no statistically significant difference was observed despite a lower mean count in tumor tissues (Fig. 1B). The majority of eccDNAs ranged from 0 to 200 bp in length, displaying a unimodal distribution (Fig. 1C, Fig. S1A-B). A volcano plot identified 5,465 upregulated and 6,466 downregulated eccDNAs in tumors (Fig. 1D). The normalized genomic coverage of eccDNAs across corresponding genomic elements (Fig. 1E) showed the lowest levels in CpG islands, with no significant differences between the two groups. The number of eccDNAs per megabase across chromosomes (Fig. 1F, Fig. S1C-D) revealed a markedly lower abundance on the Y chromosome. Additionally, the distribution of eccDNAs across chromosomes (Fig. 1G) indicated statistically significant differences at Chr2, Chr10, Chr13, Chr15, and Chr18. Further analysis (Fig. 1H) demonstrated that most eccDNAs originated from a single gene segment, though some were derived from multiple genes. Notably, different eccDNAs could be generated from the same gene depending on fragment length (Fig. 1I). Gene Ontology (GO) and Kyoto Encyclopedia of Genes and Genomes (KEGG) analyses were performed on differentially expressed eccDNA-associated genes. Among upregulated eccDNA-related genes, GO analysis (Fig. 1J) highlighted enrichment in the regulation of GTPase activity, while KEGG pathway analysis (Fig. 1K) identified significant enrichment in the Calcium signaling pathway and Axon guidance. Conversely, downregulated eccDNA-related genes were primarily associated with trans-synaptic signaling regulation (Fig. 1L), with KEGG analysis (Fig. 1M) revealing enrichment in Axon guidance.

### Detection and analysis of eccDNAs in cell lines

**Figure 2:**
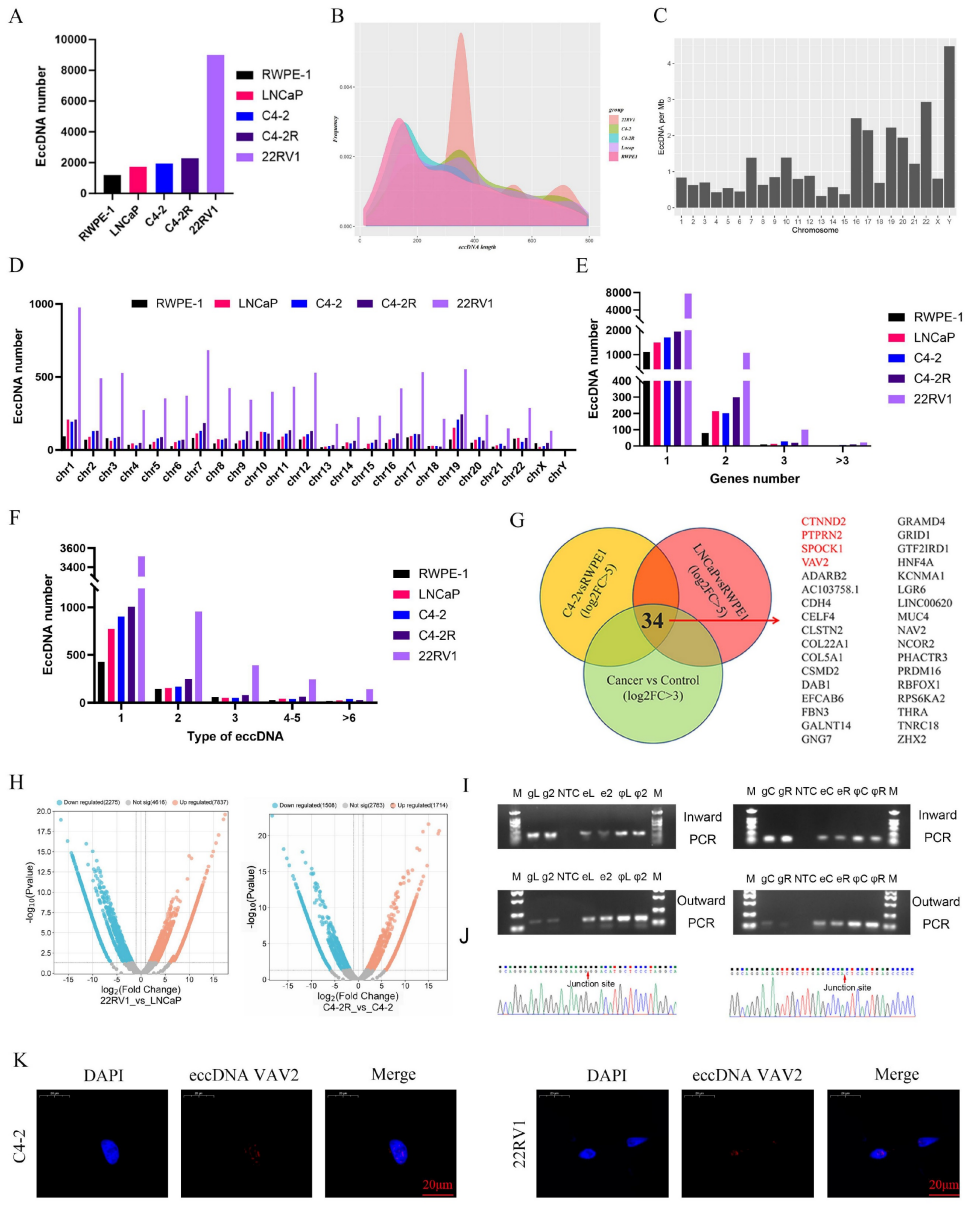
** Detection and analysis of eccDNAs in cell lines. A** The number of eccDNAs in different cell lines. **B** The distribution of eccDNAs. **C** The number of eccDNAs per Mb chromosome in RWPE-1. **D** The number of eccDNAs derived from each chromosome. **E** Number of eccDNAs derived from different genes. **F** Types of eccDNAs included different types of genes. **G** The Vennis from 3 groups. **H** Volcano plot analysis between the C4-2R vs C4-2 group and 22RV1 vs LNCaP. **I-J** The verification of eccDNA VAV2 in 22RV1 and LNCaP, C4-2R and C4-2. **K** The location of eccDNA VAV2 by FISH assay.

To further investigate the role of eccDNAs in PCa, eccDNA profiling was performed in RWPE-1 and PCa cell lines (C4-2, C4-2R, LNCaP, and 22RV1). Circle-seq analysis revealed a higher eccDNA abundance in PCa cell lines compared to RWPE-1, with 22RV1 exhibiting the highest eccDNA count (Fig. 2A). Unlike in tissue samples, eccDNA length distribution in cell lines showed a significant increase and displayed a multi-peak pattern (Fig. 2B). Notably, in RWPE-1 cells, the Y chromosome harbored the highest eccDNA density per megabase, whereas in C4-2, C4-2R, LNCaP, and 22RV1, chromosome 19 exhibited the highest eccDNA density (Fig. 2C, Fig. S2A). The total eccDNA count across chromosomes (Fig. 2D) indicated that 22RV1 consistently exhibited the highest eccDNA levels regardless of chromosomal origin. As depicted in Fig. 2E, most eccDNAs originated from a single gene segment, while some were derived from multiple genes. Additionally, eccDNA variants derived from the same gene were observed, depending on fragment length (Fig. 2F).

To elucidate the biological functions of eccDNA-associated genes, differential eccDNA expression was analyzed between C4-2/LNCaP and RWPE-1 cells using volcano plots. In the C4-2 vs. RWPE-1 comparison, 1,514 eccDNAs were upregulated, while 938 were downregulated (Fig. S2B). Similarly, in the LNCaP vs. RWPE-1 comparison, 2,139 eccDNAs exhibited increased expression, while 915 were downregulated (Fig. S2B). GO and KEGG enrichment analyses were subsequently performed for both upregulated and downregulated eccDNA-related genes (Fig. S2C-J).

Further analysis identified 34 genes shared among the upregulated eccDNA-associated genes across the three PCa cell lines (Fig. 2G). Notably, only four of these genes--CTNND2, PTPRN2, SPOCK1, and VAV2--were also upregulated in the PCa TCGA (The Cancer Genome Atlas) database (Fig. S2K). To further explore the role of eccDNAs in Enzalutamide resistance, eccDNA profiles were compared between Enzalutamide-insensitive and -sensitive cells. Differentially expressed eccDNAs and genes were screened in the "C4-2R vs. C4-2" and "22RV1 vs. LNCaP" comparisons. Notably, VAV2-derived eccDNAs exhibited significant upregulation in both resistant cell lines (Fig. 2H). Table 1 summarizes ecc-VAV2 expression across the four PCa cell lines, showing ecc-VAV2 lengths of 1,517 bp in the C4-2R vs. C4-2 group and 702 bp in the 22RV1 vs. LNCaP group. To validate the presence of VAV2 within PCa-derived eccDNAs, outward PCR, inward PCR, and Sanger sequencing were performed (Fig. 2I-J), confirming the circular structure of ecc-VAV2 and its precise junction site. To further determine the subcellular localization of ecc-VAV2, a specific probe targeting the eccDNA junction site was designed for FISH analysis. The results demonstrated predominant nuclear localization of ecc-VAV2 in PCa cells (Fig. 2K).

### VAV2 is associated with the poor prognosis of PCa

To further investigate the clinical significance of eccDNA-associated genes, the correlation between VAV2 expression and various clinical parameters was analyzed across multiple databases. TIMER2.0 analysis revealed significantly elevated VAV2 expression across multiple tumor types, including PCa (Fig. S3A). Validation using TCGA data demonstrated a positive correlation between VAV2 expression and T stage, indicating increased expression in advanced tumors (Fig. S3B). Additionally, a significant association was observed between VAV2 levels and lymph node metastasis (Fig. S3C). Given the critical role of Gleason score and PSA levels in PCa evaluation, further analysis revealed a strong positive correlation between VAV2 expression and both clinical indicators (Fig. S3D-E). Notably, high VAV2 expression was linked to poor patient prognosis. While overall survival (OS) differences in the TCGA cohort were not statistically significant (Fig. S3F), ICGC analysis demonstrated that patients with high VAV2 expression had significantly shorter OS (Fig. S3G). Moreover, elevated VAV2 levels were significantly associated with poor disease-free survival (DFS), disease-specific survival (DSS), and progression-free survival (PFS) (Fig. S3H-J).

### The expression of VAV2 in tissues and cell lines

**Figure 3:**
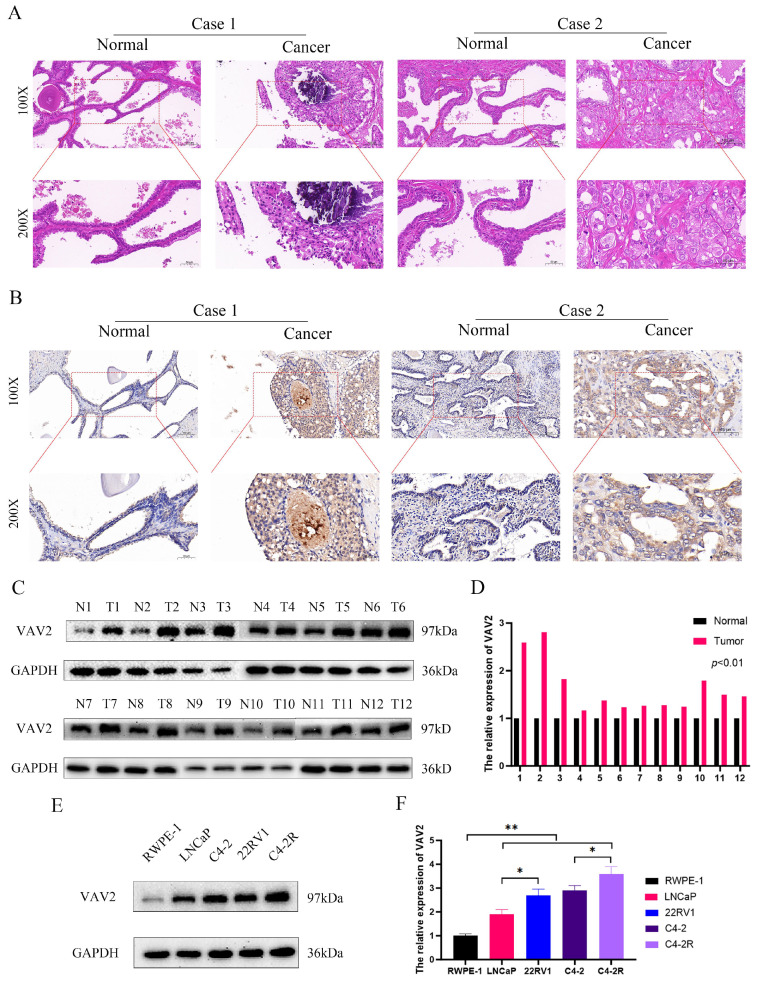
** The expression of VAV2 in tissues and cell lines. A** HE and **B** IHC showed the expression of VAV2 in PCa tissues. **C-D** The VAV2 protein level was measured by Western blot. **E**-**F** The protein expression of VAV2 in cell lines. *P < 0.05, **P < 0.01.

To further validate VAV2 expression in clinical tissues, HE staining and IHC were performed (Fig. 3A-B). IHC results confirmed a significant increase in VAV2 expression in PCa tissues. Western blot analysis further corroborated these observations, showing upregulated VAV2 levels in PCa samples (Fig. 3C-D). Notably, VAV2 expression exhibited a positive correlation with ecc-VAV2 abundance. To validate this relationship *in vitro*, VAV2 expression was assessed in PCa cell lines compared to RWPE-1. Western blot analysis demonstrated elevated VAV2 protein levels in LNCaP and C4-2 cells relative to RWPE-1 (Fig. 3E-F). Furthermore, VAV2 expression was significantly upregulated in Enzalutamide-resistant PCa cells (Fig. 3E-F), reinforcing its potential role in resistance mechanisms.

### VAV2 regulates the proliferation and metastasis of PCa cells

**Figure 4:**
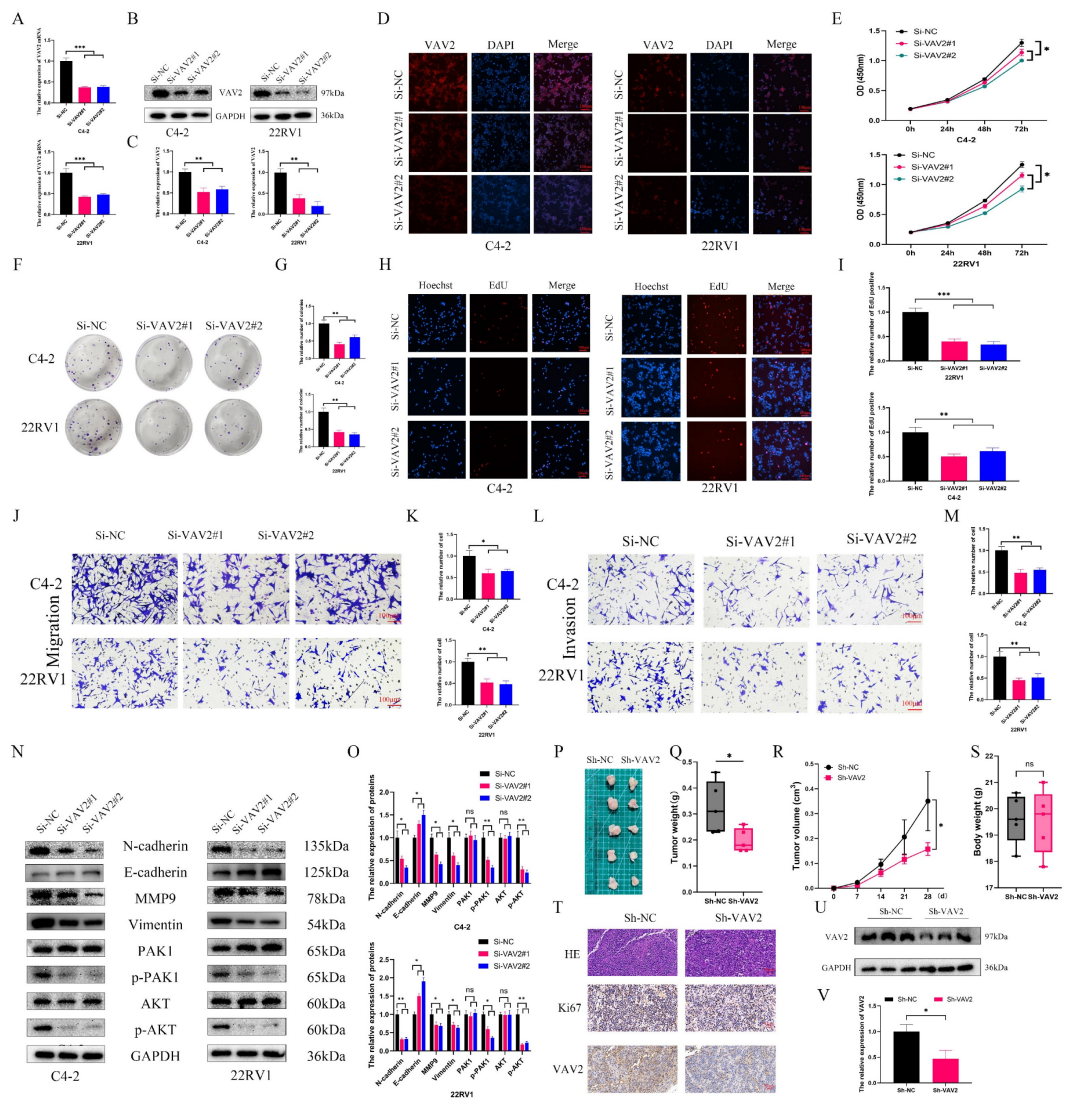
** Down-regulation of VAV2 inhibits the proliferation and metastasis of PCa cells. A** The mRNA expression of VAV2 by qRT-PCR. **B-C** The protein level of VAV2 by Western blot. **D** IF was used to measure the VAV2 protein level. **E** CCK-8 and **F-G** Colony formation assays demonstrated the proliferation of PCa cells. **H-I** EdU assay showed the decreased growth rate after knockdown of VAV2. **J-K** Cell migration and **L-M** invasion ability were measured. **N-O** Down-regulation of VAV2 inhibited the EMT process and PAK1/AKT pathway. **P** The function of VAV2 *in vivo* after C4-2 cells deletion of VAV2 or not **Q-S** The tumor weight, tumor volume curve and body weight were measured and analyzed. **T** Expression of Ki67, and VAV2 was measured in xenograft tumors HE/IHC staining. **U-V** Western blot showed the expression of VAV2 in xenograft tumors. *P < 0.05, **P < 0.01, ***P < 0.001, "ns" is not significant.

To investigate the biological role of VAV2 in PCa, loss-of-function models were established in C4-2 and 22RV1 cells. qRT-PCR confirmed a significant reduction in VAV2 expression following transfection with Si-VAV2 (Fig. 4A), and Western blot further validated the knockdown efficiency (Fig. 4B-C). IF staining demonstrated a marked decrease in fluorescence intensity after VAV2 depletion (Fig. 4D, Fig. S4). CCK-8 assays revealed a significant reduction in PCa cell proliferation upon VAV2 knockdown (Fig. 4E), which was further corroborated by colony formation assays (Fig. 4H-I). EdU assays showed a substantial decline in EdU-positive cells, indicating that VAV2 silencing significantly impaired PCa cell proliferation. Furthermore, Transwell assays demonstrated that VAV2 knockdown markedly suppressed both migration and invasion (Fig. 4J-M). Given that VAV2 has been reported to activate PAK1 *via* Rac1 to promote tumor progression^19^, ^20^, its involvement in PCa was further examined. Western blot analysis revealed a significant reduction in PAK1 and AKT phosphorylation following VAV2 knockdown (Fig. 4N-O). Additionally, EMT-related protein markers were also downregulated (Fig. 4N-O). *In vivo*, VAV2 depletion in C4-2 xenografts resulted in reduced tumor growth, as evidenced by decreased tumor volume and weight (Fig. 4P-S, Fig. S5). IHC staining and Western blot analysis further demonstrated that VAV2 and Ki67 expression levels correlated with tumor size (Fig. 4T-V).

**Figure 5:**
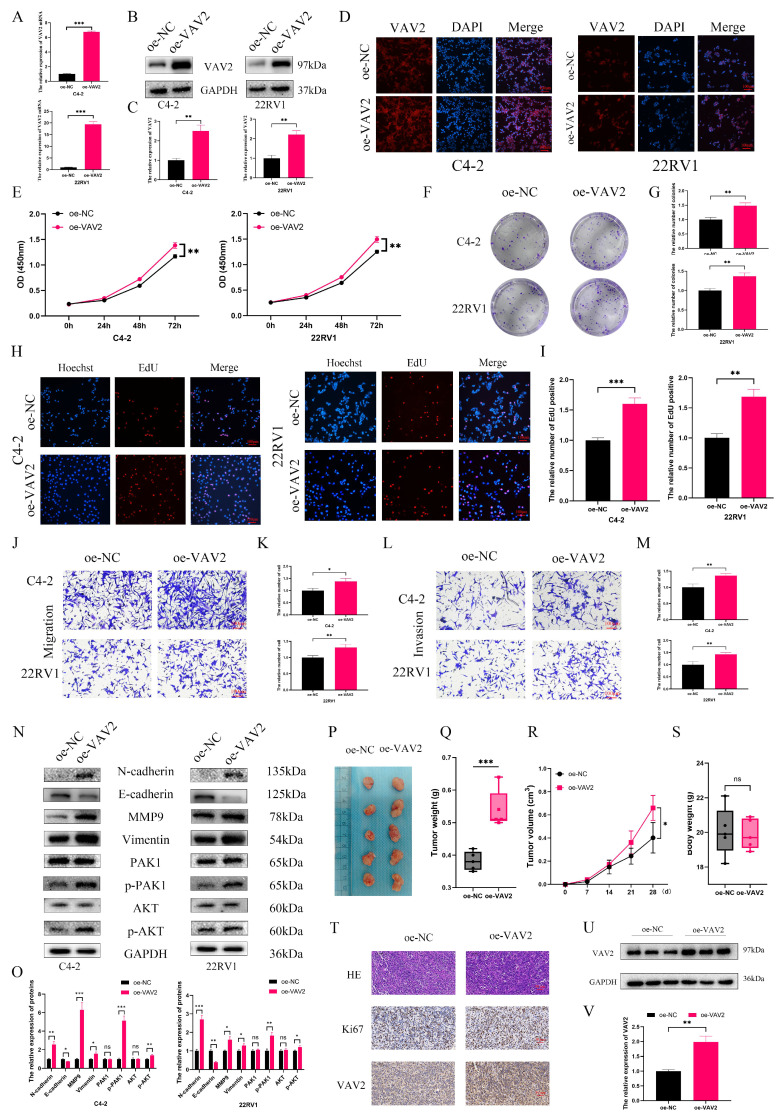
** Up-regulation of VAV2 promotes the proliferation and metastasis of PCa cells. A** The mRNA expression of VAV2 by qRT-PCR. **B-C** The protein level of VAV2 by Western blot. **D** IF was used to measure the VAV2 protein level. **E** CCK-8 and **F-G** Colony formation assays demonstrated the proliferation of PCa cells. **H-I** EdU assay showed the growth rate was promoted after overexpression of VAV2. **J-K** Cell migration and **L-M** invasion ability were measured. **N-O** Up-regulation of VAV2 promoted the EMT process and PAK1/AKT pathway. **P** The function of VAV2 *in vivo* of 22RV1 cells **Q-S** The tumor weight, tumor volume curve and body weight were measured and analyzed. **T** Expression of Ki67, and VAV2 was measured in xenograft tumors HE/IHC staining. **U-V** Western blot showed the expression of VAV2 in xenograft tumors. *P < 0.05, **P < 0.01, ***P < 0.001,"ns"is not significant.

To assess whether VAV2 overexpression exerts the opposite effect, C4-2 and 22RV1 cells were transfected with an oe-VAV2 plasmid. qPCR, Western blot, and IF staining confirmed a substantial increase in VAV2 expression post-transfection (Fig. 5A-D, Fig. S6). VAV2 overexpression significantly enhanced PCa cell proliferation (Fig. 5E-I) and increased the number of migrating and invading cells in Transwell assays (Fig. 5J-M). Furthermore, overexpression of VAV2 activated the PAK1/AKT signaling pathway and promoted EMT progression (Fig. 5N-O). These results were further corroborated in *in vivo* experiments, where VAV2 overexpression accelerated tumor growth (Fig. 5P-V).

### VAV2 mediates Enzalutamide resistance through upregulation of AR/ARv7

**Figure 6:**
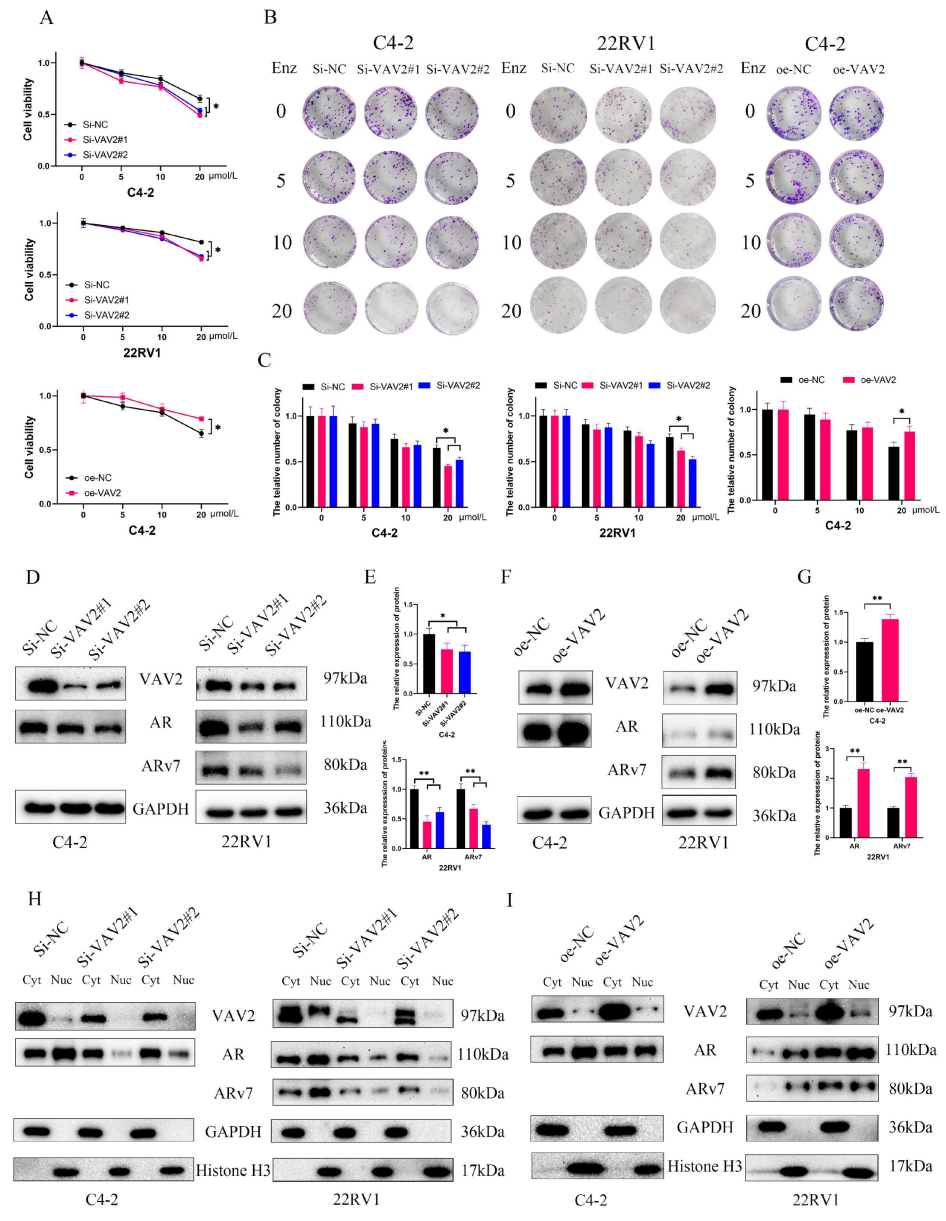
** VAV2 promotes Enzalutamide resistant of PCa *in vitro*. A** Cell viability was assessed by CCK-8 assay after being treated with Enzalutamide (0, 5, 10 and 20 μM). **B-C** Colony formation assay with VAV2 knockdown or overexpression. **D-E** Downregulation of VAV2 decreased AR/ARv7 expression. **F-G** Overexpression of VAV2 upregulated the level of AR/ARv7. **H-I** The change of VAV2 regulated the level of AR/ARv7 in the cytoplasm and nucleus. *P < 0.05, **P < 0.01.

Further investigation into the role of VAV2 in Enzalutamide resistance in PCa revealed that VAV2 knockdown increased cellular sensitivity to Enzalutamide, while VAV2 overexpression reversed this effect (Fig. 6A). Colony formation assays further demonstrated that VAV2 silencing enhanced the inhibitory effect of Enzalutamide on PCa cells, whereas VAV2 overexpression promoted colony formation despite Enzalutamide treatment (Fig. 6B-C). To elucidate the molecular mechanism underlying VAV2-mediated Enzalutamide resistance, the expression levels of AR and ARv7 were analyzed. A positive correlation between VAV2 and AR/ARv7 expression was observed, with AR/ARv7 levels increasing or decreasing in response to VAV2 overexpression or knockdown, respectively (Fig. 6D-G). Given that AR/ARv7 predominantly localizes to both the nucleus and cytoplasm, its activation of the androgen response element depends on nuclear translocation. The subcellular distribution of VAV2 was examined to determine whether it influenced AR/ARv7 localization. Western blot analysis revealed that VAV2 was primarily localized in the cytoplasm of C4-2 and 22RV1 cells, with lower expression in the nucleus. Upon VAV2 knockdown, protein levels were significantly reduced in both compartments (Fig. 6H). Although VAV2 depletion had no discernible impact on cytoplasmic AR/ARv7 expression, it markedly reduced nuclear AR/ARv7 levels (Fig. 6H). Conversely, VAV2 overexpression increased AR/ARv7 expression in both compartments, with a more pronounced effect in the cytoplasm (Fig. 6I). These results suggest that VAV2 enhances AR/ARv7 protein expression and nuclear translocation, thereby contributing to Enzalutamide resistance in PCa cells.

### VAV2 directly interacts with AR/ARv7 and regulates their stabilization

**Figure 7:**
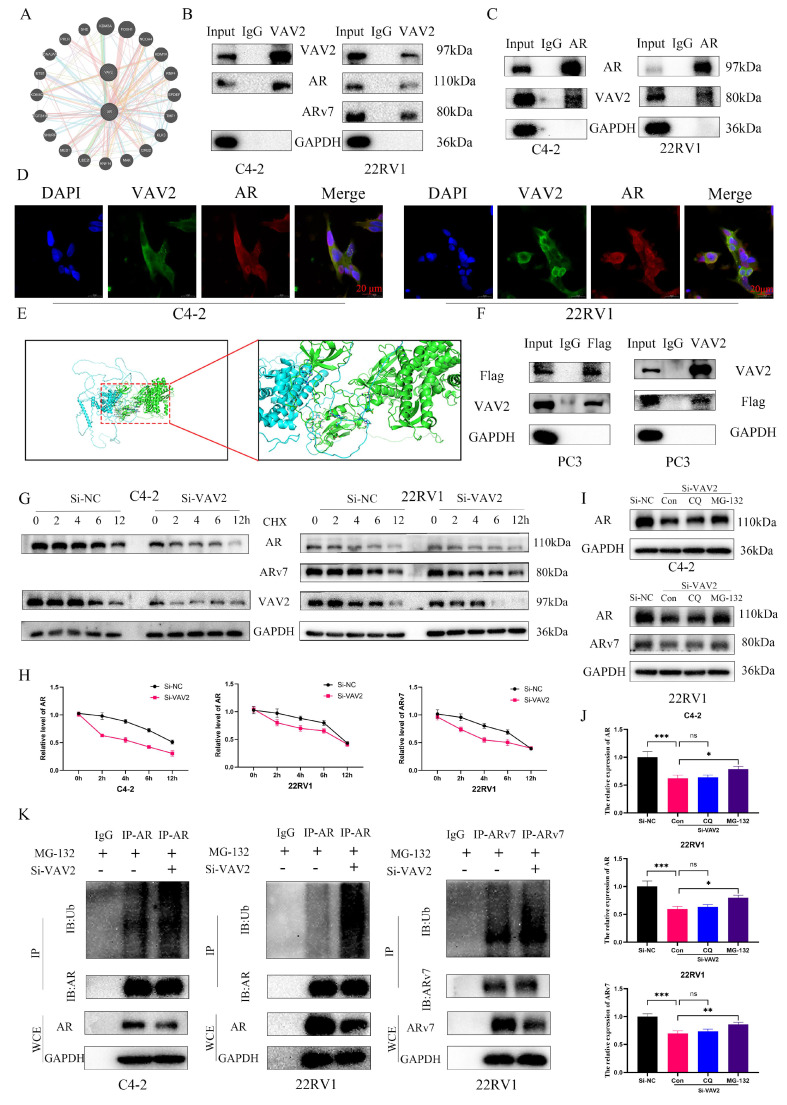
** VAV2 directly interacts with AR/ARv7 and regulates their stabilization. A** Identification the interaction between VAV2 and AR by GeneMANIA database. **B-C** Co-IP was used for examining the endogenous interaction between VAV2 and AR/ARv7. **D** Double staining for VAV2 and AR in PCa cells and was visualised by confocal microscopy. **E** The PyMOL was used to visualize protein-protein docking between VAV2 and AR. **F** Co-IP verified whether VAV2 could interact with AR-NTD. **G-H** C4-2 and 22RV1 cells with or without VAV2 knockdown were treated with CHX (50 μg/mL) and collected at 0, 2, 4, 6, and 12 hours. **I-J** Cells were transfected with VAV2 SiRNA for 24 hours and then treated with DMSO, Chloroquine (200 μM), or MG-132 (10 μM) for additional 24 hours. **K** Cells with or without VAV2 depletion were treated with MG-132 (10 μM) for 24 hours and lysed for IP and Western blot. *P < 0.05, **P < 0.01, ***P < 0.001,"ns"is not significant.

Since VAV2 knockdown reduced AR/ARv7 protein levels without altering their mRNA expression (Fig. S7), it is hypothesized that VAV2 regulates AR/ARv7 protein stabilization *via* direct interaction. The GeneMANIA database predicted a potential interaction between VAV2 and AR (Fig. 7A). To validate this, Co-IP assays were performed in C4-2 and 22RV1 cells, confirming that VAV2 directly interacts with AR to form a protein complex (Fig. 7B). Additionally, co-IP results demonstrated that VAV2 also binds directly to ARv7 (Fig. 7B). To further substantiate this interaction, a reverse co-IP assay was conducted using AR as the immunoprecipitated protein, which successfully pulled down VAV2, reinforcing the direct interaction hypothesis (Fig. 7C). Confocal immunofluorescence imaging further confirmed the co-localization of endogenous VAV2 and AR in PCa cells (Fig. 7D). Structurally, AR consists of three key domains: the DNA-binding domain (DBD), the N-terminal transcriptional activation domain (NTD), and the ligand-binding domain (LBD).

Notably, ARv7 lacks the LBD. To investigate the specific binding site, protein-protein docking simulations were conducted using PyMOL, revealing that VAV2 predominantly interacts with the NTD of AR (Fig. 7E). To experimentally confirm this, a Flag-tagged AR-NTD plasmid was constructed and transfected into AR-null PC3 cells. Subsequent co-IP assays validated that VAV2 directly interacts with the AR-NTD domain (Fig. 7F). To explore the mechanism by which VAV2 regulates AR/ARv7 protein stability, protein half-life was assessed using cycloheximide (CHX), an inhibitor of protein synthesis. VAV2 depletion significantly accelerated AR and ARv7 degradation in PCa cells (Fig. 7G-H). Given that AR/ARv7 degradation is primarily mediated by the ubiquitin-proteasome pathway, whether VAV2 stabilizes AR/ARv7 by modulating their ubiquitination status was investigated. Cells with VAV2 knockdown were treated with the proteasome inhibitor MG-132 and the lysosomal inhibitor chloroquine. Western blot analysis indicated that MG-132, but not chloroquine, rescued VAV2 knockdown-induced AR/ARv7 degradation (Fig. 7I-J), suggesting proteasomal involvement. Further analysis of AR/ARv7 ubiquitination revealed that VAV2 depletion significantly increased AR and ARv7 ubiquitination levels (Fig. 7K). These results indicate that VAV2 enhances Enzalutamide resistance in PCa by stabilizing AR/ARv7 protein levels.

**Figure 8:**
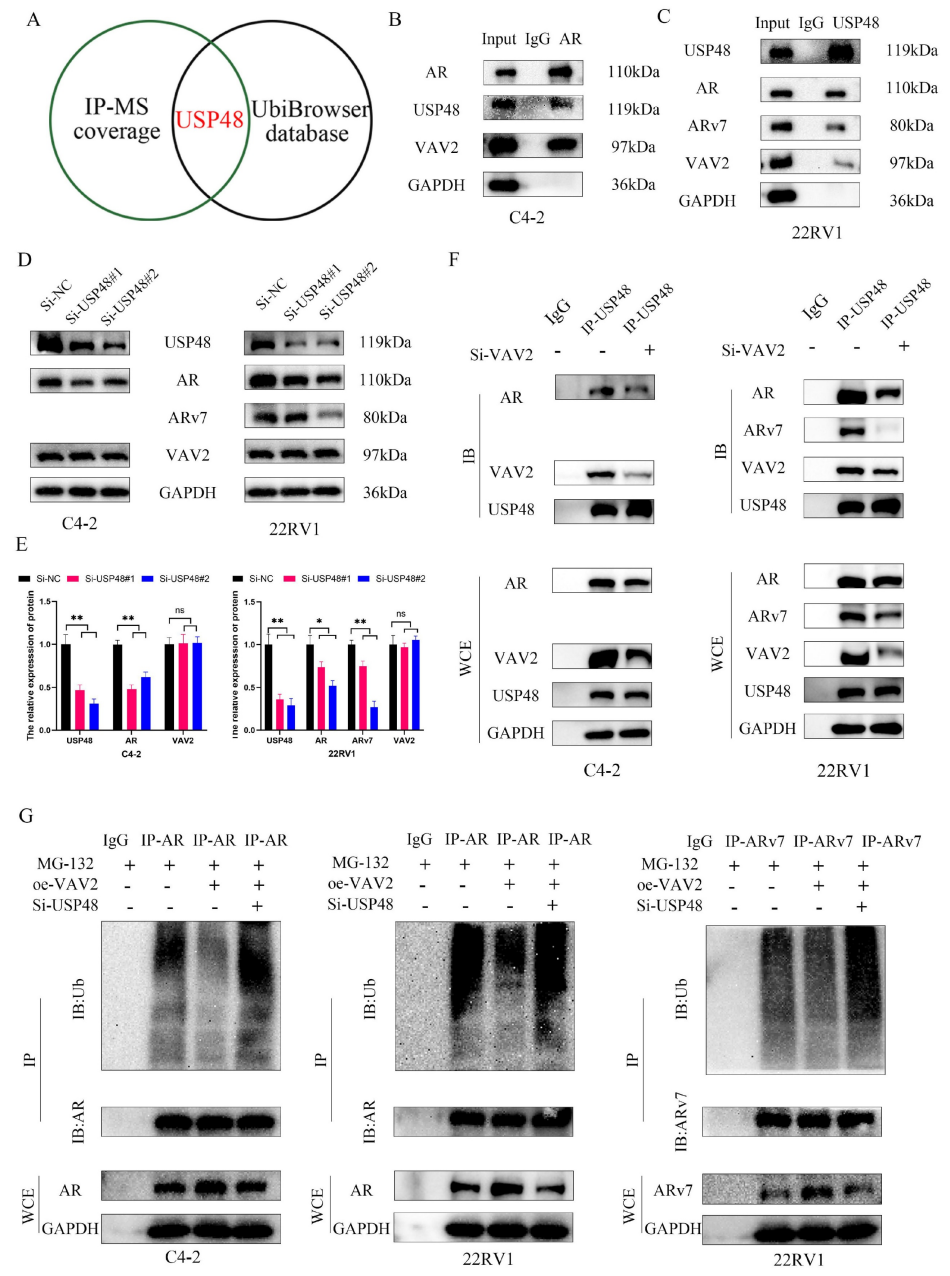
** VAV2 regulates AR/ARv7 deubiquitination *via* recruiting USP48. A** Identification of interacting proteins with VAV2 *via* mass spectrometry and deubiquitinase proteins of AR *via* UbiBrowser 2.0 database (http://ubibrowser.bio-it.cn/ubibrowser_v3/). **B-C** Co-IP was used for examining the endogenous interaction between USP48, VAV2 and AR/ARv7 in C4-2 and 22RV1 cells. **D-E** The effect of USP48 depletion on the levels of VAV2 and AR/ARv7 proteins was investigated. **F** Effect of VAV2 knockdown on the interaction between USP48 and AR/ARv7. **G** The effects of VAV2 overexpression or USP48 depletion on AR/ARv7 ubiquitination levels. *P < 0.05, **P < 0.01,"ns"is not significant.

To elucidate the precise mechanism by which VAV2 decreases AR/ARv7 ubiquitination, mass spectrometry analysis (Table S1) and UbiBrowser 2.0 were utilized to predict potential AR-associated deubiquitinases (Table S2). Among the identified candidates, ubiquitin-specific peptidase 48 (USP48) was the only protein common to both datasets (Fig. 8A). Co-IP assays confirmed direct interactions between USP48 and both VAV2 and AR/ARv7 (Fig. 8B-C). Furthermore, USP48 knockdown significantly reduced AR/ARv7 protein levels without affecting VAV2 expression (Fig. 8D-E). To further investigate how VAV2 regulates AR/ARv7 stabilization, the interaction between AR/ARv7 and USP48 was assessed following VAV2 depletion. Co-IP results demonstrated that VAV2 knockdown weakened the interaction between USP48 and AR/ARv7 (Fig. 8F). Ubiquitination assays revealed that VAV2 overexpression attenuated AR/ARv7 ubiquitination; however, this effect was reversed upon USP48 depletion (Fig. 8G). These results collectively demonstrate that VAV2 stabilizes AR/ARv7 by enhancing the association between USP48 and AR/ARv7, thereby promoting Enzalutamide resistance.

### VAV2 decreases the sensitive of PCa cells to Enzalutamide *in vivo*

**Figure 9:**
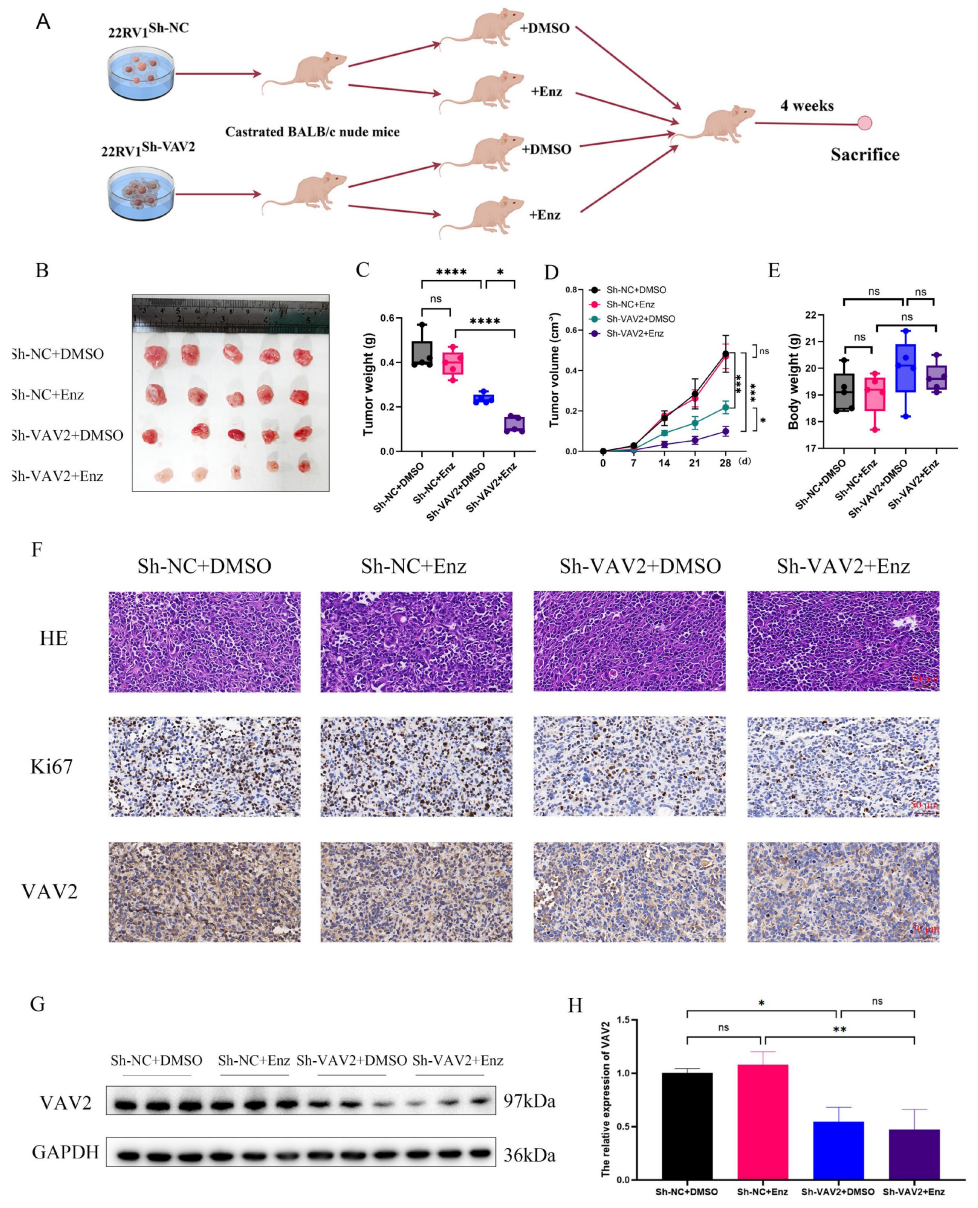
** VAV2 decreases the sensitive of PCa cells to Enzalutamide *in vivo*. A-B** Mice bearing 22RV1 xenografts were treated with DMSO or enzalutamide (10 mg/kg p.o.) or their combination for 4 weeks (n = 5/group) with or without VAV2 depletion, and all xenografts in each group was photographed, and schematic diagram drawn by figdraw. **C-E** The tumor weight, tumor volume curve and body weight were measured and analyzed. **F** Expression of Ki67, and VAV2 was measured in xenograft tumors HE/IHC staining. **G-H** Western blot showed the expression of VAV2 in xenograft tumors. *P < 0.05, **P < 0.01, ****P < 0.0001,"ns"is not significant.

To evaluate the therapeutic potential of targeting VAV2 in PCa xenografts, subcutaneous implantation of 22RV1 cells with or without VAV2 knockdown was performed in castrated mice. Mice were randomly assigned to four groups: Sh-NC+DMSO, Sh-NC+Enz, Sh-VAV2+DMSO, and Sh-VAV2+Enz. Tumor growth was significantly inhibited in the VAV2-knockdown group, with further suppression observed upon Enzalutamide treatment (Fig. 9A-B). Importantly, no significant differences in body weight were observed across groups. Tumor weight and volume were markedly reduced following VAV2 knockdown, with the most pronounced reduction observed in the VAV2-knockdown group treated with Enzalutamide (Fig. 9C-D). IHC analysis further supported this observation, as Ki67-positive cell counts were lower in the Sh-VAV2+DMSO group compared to Sh-NC+DMSO, and further reduced following Enzalutamide treatment (Fig. 9E). Notably, Enzalutamide monotherapy did not significantly impact tumor growth compared to the control group. Western blot analysis confirmed that VAV2 expression remained unchanged between the Sh-NC+DMSO and Sh-NC+Enz groups, as well as between the Sh-VAV2+DMSO and Sh-VAV2+Enz groups, but was significantly downregulated in VAV2-knockdown tumors (Fig. 9F-G). Collectively, these results suggest that VAV2 promotes PCa progression by activating the PAK1/AKT signaling pathway while simultaneously stabilizing AR/ARv7 proteins, sustaining their function even in the presence of Enzalutamide.

### VAV2 is transcriptionally regulated by AR in PCa cells

**Figure 10:**
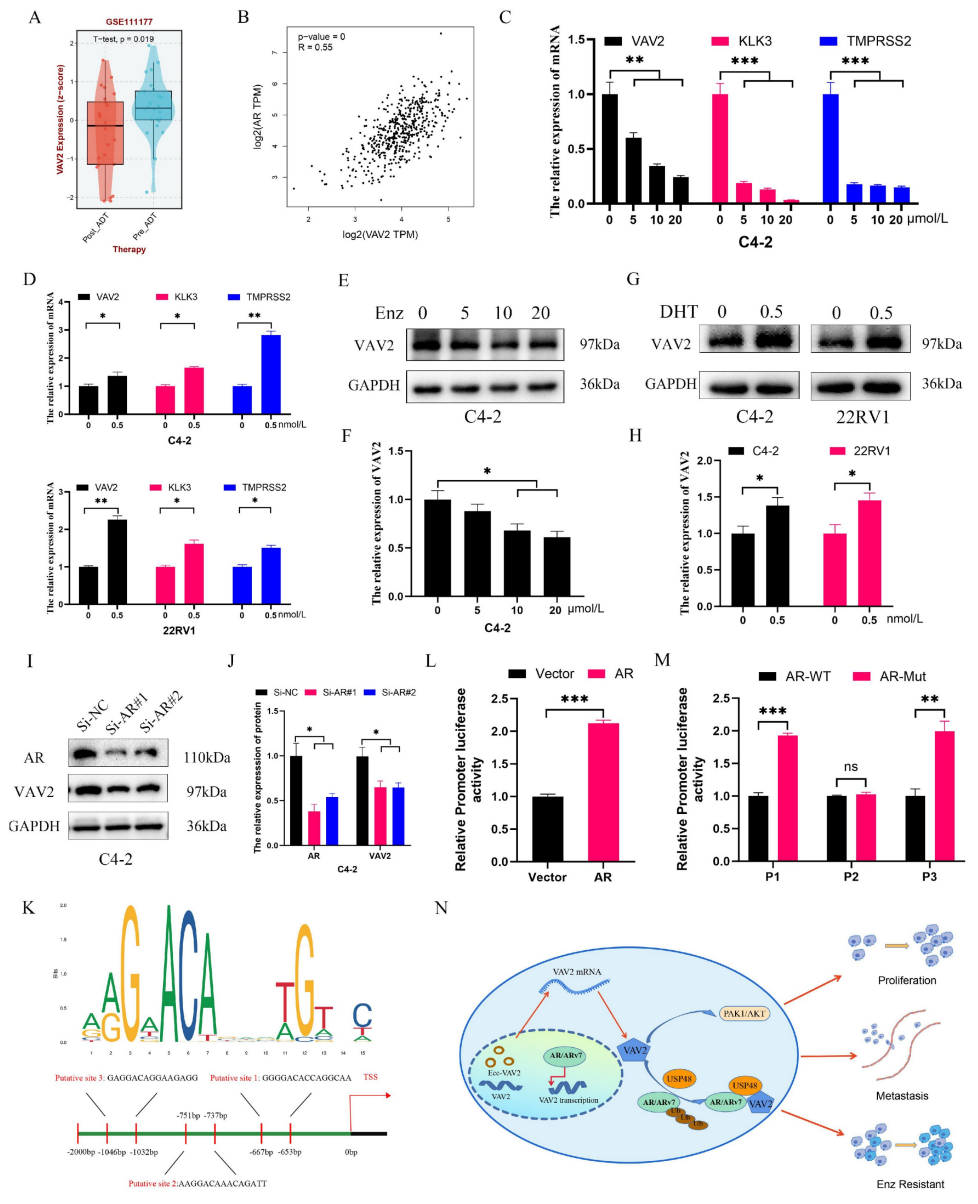
** AR regulates VAV2 transcription in PCa cells. A** The level of VAV2 mRNA in PCa patients after receiving ADT treatment. **B** The correlation of AR and VAV2 in GEPIA database (http://gepia2.cancer-pku.cn/#index).** C** The relation expression of VAV2 mRNA after treatment with Enzalutamide (0, 5, 10 and 20 μM). **D** The relation expression of VAV2 mRNA after treatment with DHT (0.5 M). **E-F** The relation expression of VAV2 protein after treatment with Enzalutamide (0, 5, 10 and 20 μM) in C4-2 cells. **G-H** The relation expression of VAV2 protein after treatment with DHT (0.5 nM). **I-J** The expression of VAV2 after downregulation of AR. **K** The possible binding sites of AR on the VAV2 promoter with the Jaspar database (https://jaspar.elixir.no/). **L** Double luciferase assay in C4-2 cells after transfection of AR. **M** Double luciferase assay in C4-2 cells after transfection of wild AR or truncated mutated AR for each putative position. **N** The positive feedback signaling pathway on how VAV2 promotes Enzalutamide resistant of PCa cells. *P < 0.05, **P < 0.01, ***P < 0.001,"ns"is not significant.

As a transcription factor, AR was hypothesized to facilitate VAV2 transcription. Analysis of the GSE111177 dataset revealed a significant reduction in VAV2 mRNA levels in patients with PCa following ADT treatment (Fig. 10A), with a positive correlation between AR and VAV2 expression (Fig. 10B). IHC and Western blot analyses further confirmed that AR expression was markedly elevated in tumors, aligning with VAV2 expression patterns (Fig. S8). Enzalutamide treatment resulted in a dose-dependent decrease in VAV2 mRNA levels (Fig. 10C). In C4-2 and 22RV1 cells cultured in charcoal-stripped medium for 48 h and subsequently treated with DHT, VAV2 mRNA expression was significantly upregulated (Fig. 10D), consistent with Western blot findings (Fig. 10E-H). AR knockdown in C4-2 cells led to a substantial reduction in both VAV2 mRNA and protein levels (Fig. 10I-J). To determine whether AR directly binds to the VAV2 promoter to enhance transcription, potential AR binding sites were predicted using the JASPAR database, identifying three candidate sequences: P1 (-653 to -667), P2 (-737 to -751), and P3 (-1,032 to -1,046) (Fig. 10K). Luciferase assays in C4-2 cells transfected with AR confirmed a significant activation of VAV2 transcriptional activity compared to the wild-type control (Fig. 10L). To determine the specific AR binding site responsible for VAV2 transcriptional regulation, truncation mutations were introduced at three predicted sites within the VAV2 promoter. Among them, only the P2 truncation mutation significantly suppressed VAV2 transcriptional activity, indicating that AR is predominantly recruited to P2, rather than P1 or P3, to facilitate VAV2 transcription (Fig. 10M). ChIP assay results corroborated these findings, aligning with the luciferase assay data (Fig. S9). Collectively, these results confirm that AR directly binds to the VAV2 promoter to modulate its transcription. Based on these observations, a positive feedback loop was proposed, illustrating how VAV2 contributes to Enzalutamide resistance in PCa cells (Fig. 10N).

In summary, aberrant expression of ecc-VAV2 in PCa is associated with a marked upregulation of VAV2 transcription, which drives Enzalutamide resistance by facilitating AR/ARv7 deubiquitination through USP48 recruitment. In turn, AR enhances VAV2 transcription, further amplifying VAV2 expression.

## Discussion

PCa ranks as the second most prevalent malignancy among males worldwide. In recent years, its incidence in China has risen steadily, driven by improved living standards and the widespread implementation of screening programs^2^, ^3^. Androgen deprivation therapy (ADT) remains the cornerstone of clinical management^21^, ^22^. Second-generation anti-androgens, including Enzalutamide and Abiraterone, are primary therapeutic agents that function by inhibiting AR activity, thereby arresting cell cycle progression and triggering signaling pathways that culminate in PCa cell death^23^. While these treatments temporarily delay disease progression and extend patient survival, nearly all cases eventually advance to castration-resistant prostate cancer (CRPC)^24^. Extensive research has identified multiple signaling pathways involved in the development of Enzalutamide resistance, with AR splice variant generation being a key mechanism^25^, ^26^.

EccDNAs constitute a distinct class of circular DNA molecules that exist independently of linear chromosomes. Unlike chromosomal DNA, eccDNAs exhibit a circular conformation, demonstrate relative stability, and show resistance to nuclease degradation^8^, ^9^. Studies have confirmed their widespread presence across eukaryotic cells, with recent investigations identifying over 100,000 eccDNA species in human muscle and blood cells, carrying substantial genetic content^11^. Despite their abundance, the functional significance of eccDNAs remains poorly understood due to limited research. However, accumulating evidence highlights their potent mutagenic potential in tumor cells, significantly promoting tumorigenesis and disease progression^27^-^29^. Yang et al. demonstrated that PLCG2-derived eccDNA facilitates lung cancer metastasis by regulating mitochondrial respiration^30^. Similarly, eccDNA containing the miR-17-92 cluster plays a pivotal role in hepatocarcinogenesis^31^. Furthermore, eccDNAs have been implicated in drug resistance across various malignancies. For instance, eccDNA-mediated MUC20 regulation reduces multiple myeloma resistance to proteasome inhibitors by inhibiting apoptosis^32^. Additionally, RAB3B-containing eccDNA contributes to cisplatin resistance by modulating autophagy in hypopharyngeal squamous cell carcinoma^33^. Notably, eccDNAs are also present in extracellular fluids, positioning them as promising biomarkers for early tumor detection^34^, ^35^. Despite these findings, studies on eccDNAs in PCa remain scarce. Circle-seq analysis confirmed their widespread presence in PCa tissues and cell lines. The data further revealed that each eccDNA could harbor distinct gene fragments and that a single gene may generate multiple eccDNA variants due to different break sites. Although no statistically significant differences were observed in eccDNA abundance among tissue samples, PCa cell lines exhibited a marked increase, with the lowest levels in LNCaP and the highest in 22RV1. To elucidate the functional implications of these eccDNAs, GO and KEGG analyses were performed on differentially expressed eccDNA-associated genes to identify potential regulatory mechanisms.

VAV2, a member of the Rho family, functions as a guanine nucleotide exchange factor that regulates small GTPases such as Rac1, RhoA, and Cdc42, key mediators of intracellular signaling pathways^15^, ^16^. Aberrant VAV2 expression or mutations are implicated in the pathogenesis of various diseases, particularly cancer. Studies have demonstrated its critical role in controlling cell proliferation, metastasis, and immune-inflammatory responses^17^-^19^, ^36^. In this study, a notable increase in VAV2-derived eccDNA was observed in both PCa tissues and cell lines. Subsequent validation identified ecc-VAV2 as a key target gene. Inward and outward PCR assays confirmed its circular structure, while Sanger sequencing verified its splicing site consistency with Circle-seq results. Given VAV2's oncogenic potential, its expression was further examined in PCa. IHC and Western blot analyses revealed a significant upregulation of VAV2 in PCa tissues. Additionally, bioinformatics analysis indicated that elevated VAV2 expression correlated with poor clinical prognosis in patients with PCa. Mechanistically, VAV2 was found to drive PCa proliferation and metastasis *via* the PAK1/AKT signaling pathway. Comparative analysis of Enzalutamide-sensitive (C4-2, LNCaP) and Enzalutamide-resistant (C4-2R, 22RV1) cell lines revealed a further increase in ecc-VAV2 expression in the resistant lines, with VAV2 expression levels mirroring ecc-VAV2 abundance. Further investigations suggested that VAV2 contributes to Enzalutamide resistance by directly interacting with AR/ARv7. Protein-protein docking analysis using PyMOL predicted VAV2 binding to the NTD domain of AR. To validate this interaction, Flag-AR-NTD was constructed and transfected into AR-null PC3 cells, and co-IP assays confirmed VAV2-AR-NTD binding. Additionally, VAV2 was detected in both the cytoplasm and nucleus, where it modulated AR distribution in PCa cells.

Extensive research has demonstrated that the ubiquitin-proteasome system plays a pivotal role in AR/ARv7 protein degradation^37^-^39^. To investigate how VAV2 regulates AR/ARv7 expression, protein half-life analysis revealed that VAV2 depletion significantly shortened the half-life of AR/ARv7 in PCa cells. Western blot analysis showed that MG-132, but not Chloroquine, reversed VAV2 depletion-induced AR/ARv7 downregulation, suggesting proteasomal degradation as the primary mechanism. Additionally, ubiquitination assays confirmed that VAV2 depletion enhanced AR/ARv7 ubiquitination. To elucidate the mechanism underlying VAV2-mediated AR/ARv7 deubiquitination, mass spectrometry and UbiBrowser 2.0 analysis were employed to predict potential deubiquitinases. USP48 emerged as the only candidate common to both analyses, and co-IP assays confirmed interactions between USP48, VAV2, and AR/ARv7. Further experiments demonstrated that VAV2 reduces AR/ARv7 ubiquitination by recruiting USP48. Bioinformatics analysis of the GEO dataset revealed a significant reduction in VAV2 mRNA levels in patients with PCa undergoing ADT, with a positive correlation between AR and VAV2 expression. Functional validation experiments confirmed that VAV2 transcription was suppressed by Enzalutamide and activated by DHT in PCa cells. Promoter mutation assays identified P2 as the primary AR binding site responsible for VAV2 transcriptional regulation. In summary, aberrant ecc-VAV2 expression in PCa is associated with increased VAV2 transcription, which enhances Enzalutamide resistance by facilitating AR/ARv7 deubiquitination through USP48 recruitment. In turn, AR promotes VAV2 transcription, establishing a positive feedback loop that exacerbates resistance.

However, this study has several limitations. First, changes in ecc-VAV2 expression before and after the development of Enzalutamide resistance could not be assessed due to the unavailability of matched tissue specimens. Second, while ecc-VAV2 presence in PCa was confirmed *via* outward PCR, inward PCR, Sanger sequencing, and FISH, the precise mechanism by which ecc-VAV2 regulates VAV2 expression remains unclear. Finally, although the function of VAV2 was validated in xenograft mouse models, these models do not fully capture the biological heterogeneity of patients with PCa. Potential off-target effects may further limit the clinical translatability of these findings.

## Conclusion

In conclusion, this study provides the first comprehensive characterization of eccDNAs in PCa tissues and cell lines, detailing their abundance, length distribution, genomic origin, gene localization, differential expression, and pathway enrichment by analyzing eccDNA-associated genes. To elucidate the role of eccDNA in Enzalutamide resistance, androgen-sensitive and androgen-resistant cell lines were compared, revealing that VAV2 exists in both linear and circular DNA forms. Functional analyses demonstrated that VAV2 drives PCa proliferation and metastasis *via* the PAK1/AKT signaling pathway and promotes Enzalutamide resistance by stabilizing AR/ARv7 proteins through USP48 recruitment. Furthermore, the AR-VAV2 positive feedback loop enhances VAV2 transcription, further reinforcing this resistance mechanism. These findings establish VAV2-containing eccDNA as a tumor-promoting factor and highlight its potential as a novel biomarker for PCa detection and prognosis.

## Supplementary Material

Supplementary figures and tables.

## Figures and Tables

**Table 1:** The information of eccDNA VAV2 in PCa cells

	Chr	Location	Location	Length	Dataset
	**Chr**	**Start**	**End**	**Length**	**Dataset**
**eccDNA_16877**	Chr9	133936335	133939851	3517	C4-2R vs C4-2
**eccDNA_16865**	Chr9	133798511	133799212	702	22RV1 vs LNCaP

## Abbreviations

PCa: Prostate cancer
EccDNAs: Extrachromosomal circular DNAs
VAV2: VAV guanine nucleotide exchange factor 2
qRT-PCR: Quantitative Real-time PCR
ChIP: Chromatin immunoprecipitation
FISH: Fluorescence in Situ Hybridization
Co-IP: Co-Immunoprecipitation
IF: Immunofluorescence
HE: Hematoxylin-Eosin
IHC: Immunohistochemical
GO: Gene Ontology
KEGG: Kyoto Encyclopedia of Genes and Genomes
TCGA: The Cancer Genome Atlas
GEO: Gene Expression Omnibus
DBD: DNA-binding region
NTD: N-terminal transcriptional activation region
LBD: Ligand-binding region
USP48: Ubiquitin specific peptidase 48
CHX: Cycloheximide
DHT: Dihydrotestosterone
ADT: Androgen deprivation therapy
CRPC: Castration-resistant prostate cancer
