# Supplementary material for: VAV2 exists in extrachromosomal circular DNA and contributes Enzalutamide resistance of prostate cancer via stabilization of AR/ARv7
Source: Int J Biol Sci. 2025 Apr 9;21(6):2843–63. doi: 10.7150/ijbs.109271 (PMC12035891; doi:10.7150/ijbs.109271)
Supplement: Supplementary file 1 — Supplementary figures and tables. [file ijbsv21p2843s1.zip › supplementary materials/Supplementary Figures.pdf]

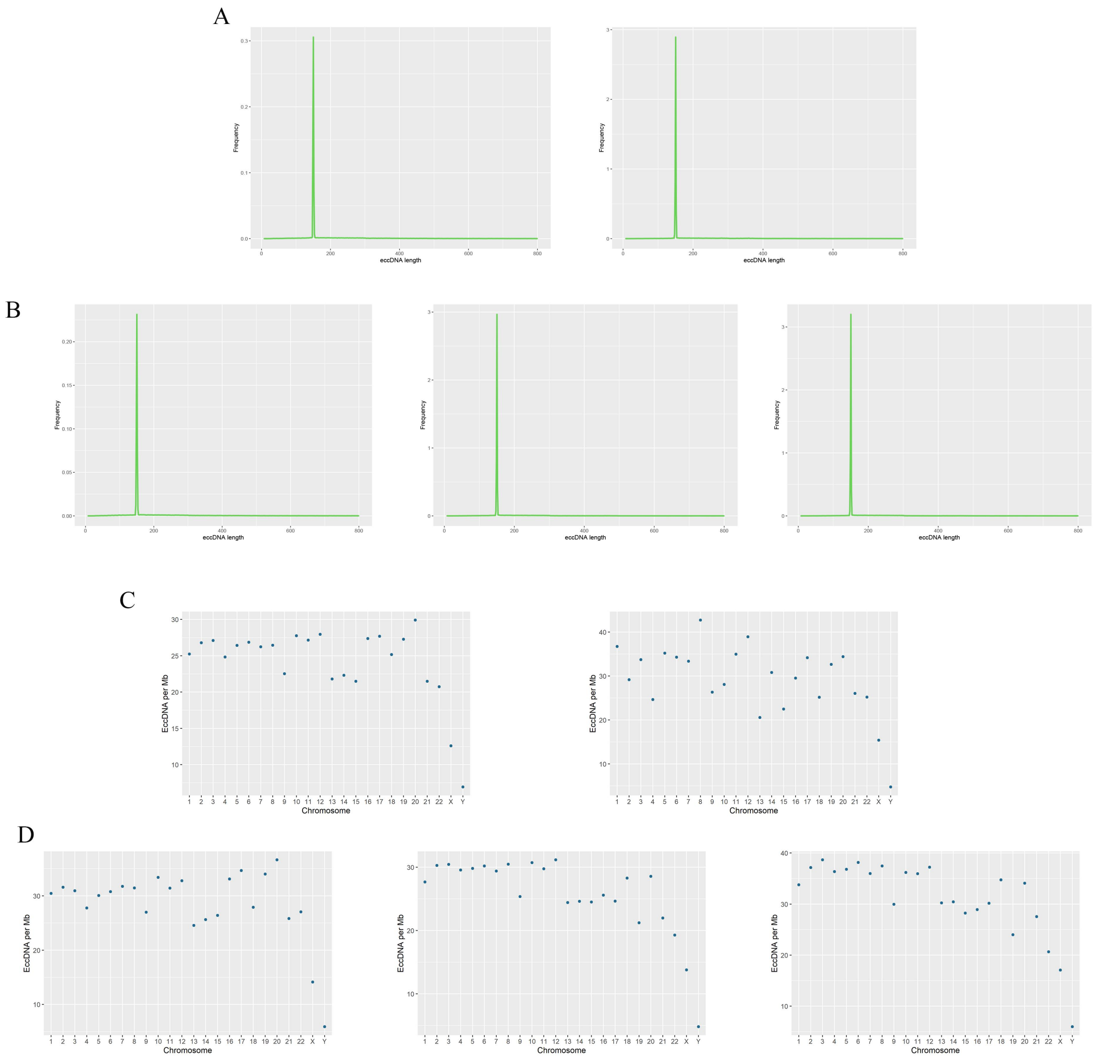

Fig. S1 The distribution of eccDNAs in tissues. **A** The tumor tissues and **B** and normal tissues distribution of eccDNAs. **C** The tumor tissues and **D** and normal tissues normalized genomic coverage of eccDNAs across corresponding genomic elements.

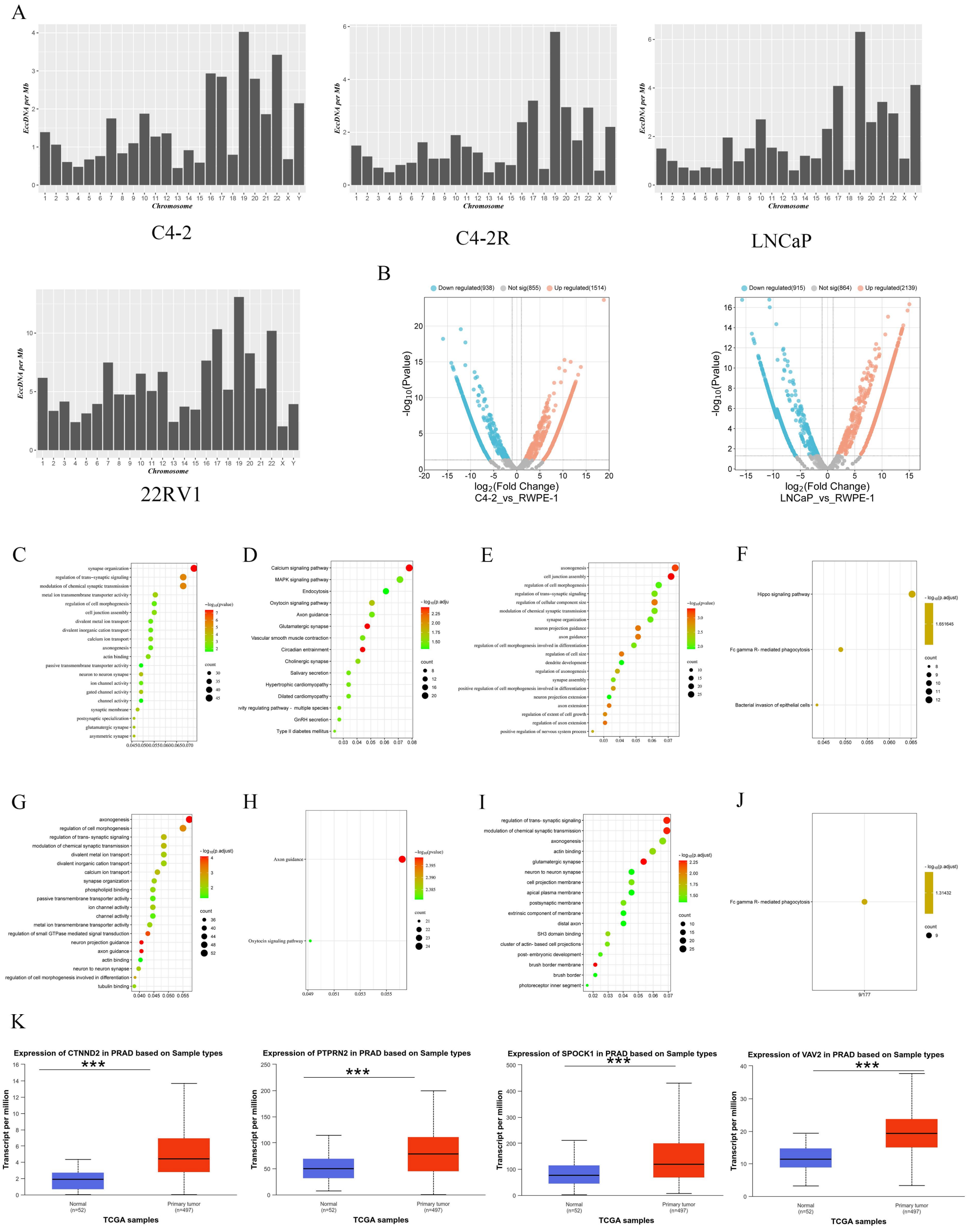

Fig. S2 The eccDNAs features and associated genes expression. **A** The number of eccDNAs per Mb chromosome in RWPE-1, C4-2, C4-2R, LNCaP and 22RV1. **B** The volcano plot analysis between the C4-2 vs RWPE-1 group and LNCaP vs RWPE-1. **C** GO and **D** KEGG enrichment analysis for the up-regulated eccDNA-related genes of the C4-2 vs RWPE-1 group. **E** GO and **F** KEGG enrichment analysis for the down-regulated eccDNA-related genes of the C4-2 vs RWPE-1 group. **G** GO and **H** KEGG enrichment analysis for the up-regulated eccDNA-related genes of the LNCaP vs RWPE-1 group. **I** GO and **J** KEGG enrichment analysis for the down-regulated eccDNA-related genes of the LNCaP vs RWPE-1 group. **K** The expression of CTNND2, PTPRN2, SPOCK1 and VAV2 from UALCAN (https://ualcan.path.uab.edu/index.html). ****P* < 0.001.

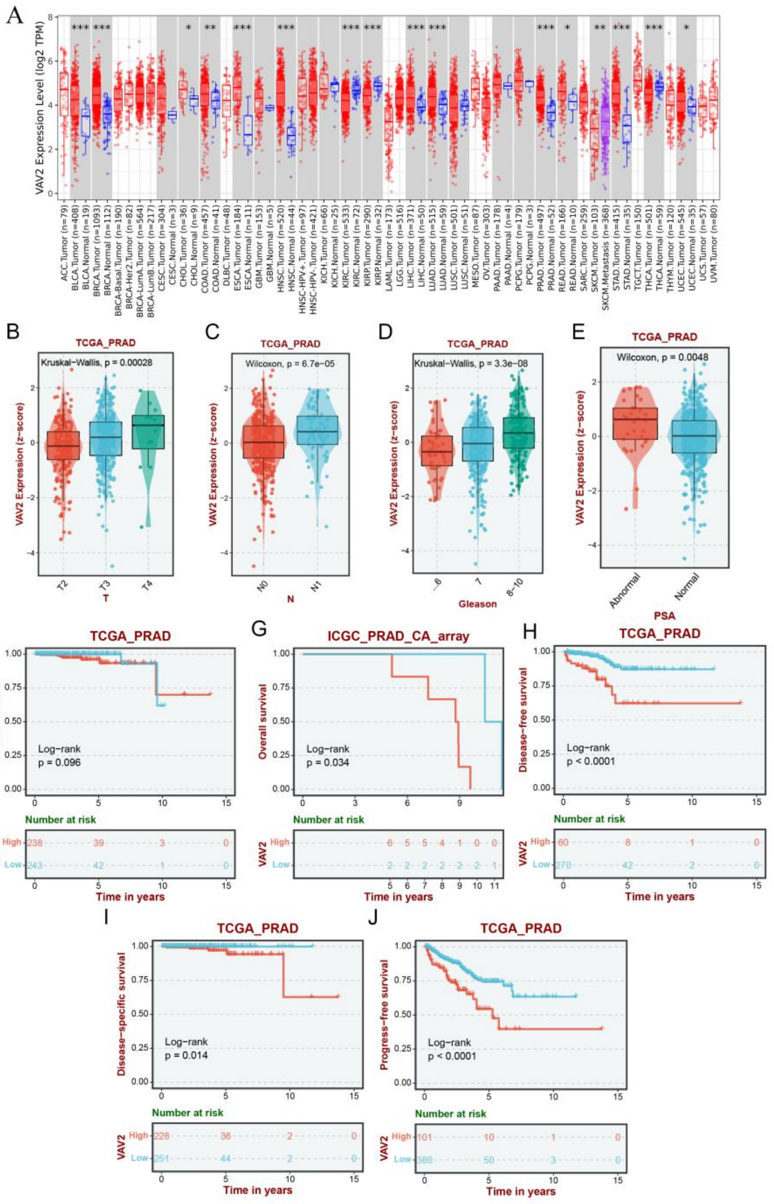

Fig. S3 VAV2 is upregulated in PCa and correlates with the poor prognosis of PCa. A Pan-cancer analysis of VAV2 in the TIMER.2.0 database (http://timer.comp-genomics.org/). **B-E** The relationship between VAV2 and PCa T stage, N stage, Gleason score, PSA level and tumor metastasis in BEST database (https://rookieutopia.hiplot.com.cn/app_direct/BEST/). **F-J** The relationship between VAV2 and PCa patients prognosis in BEST database. $*P < 0.05$, $**P < 0.01$, $***P < 0.001$.

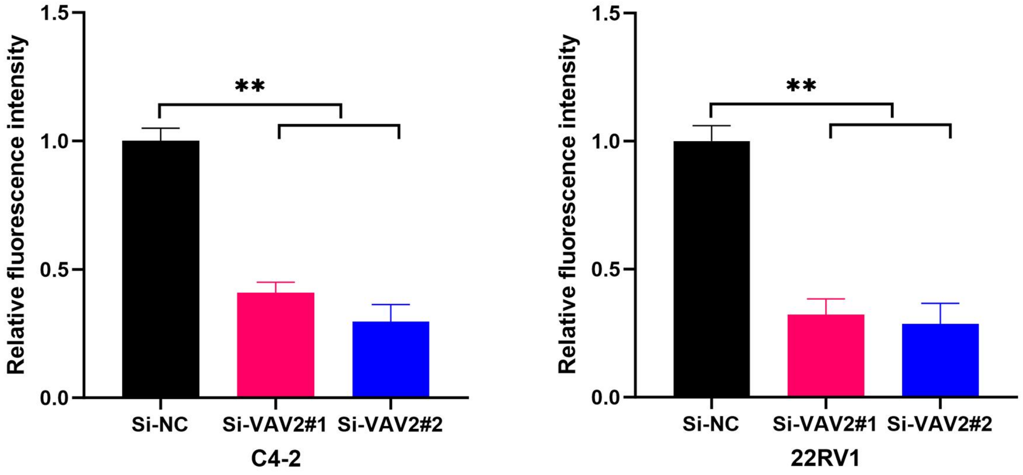

Fig. S4 The relative fluorescence intensity of VAV2 expression.

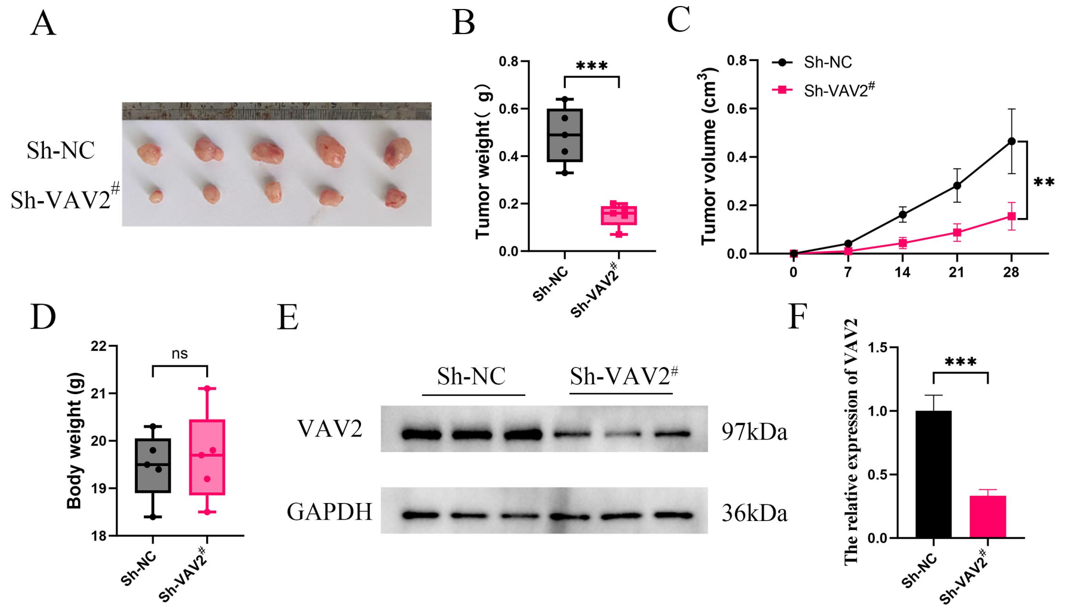

Fig. S5 Silencing VAV2 inhibits the proliferation of PCa cells in vivo. **A** The function of VAV2 in vivo of C4-2 cells. **B-D** The tumor weight, tumor volume curve and body weight were measured and analyzed. **E-F** The expression of VAV2 in different groups.

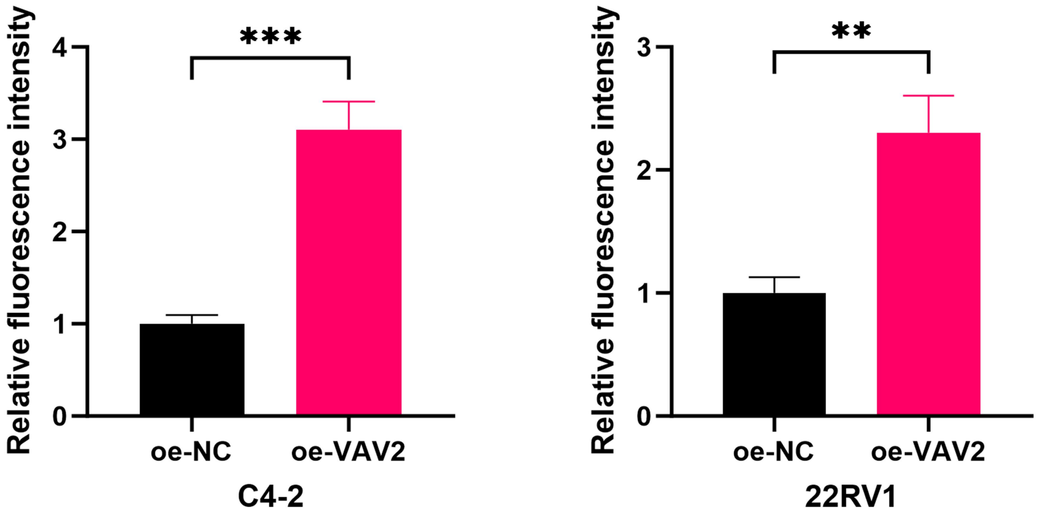

Fig. S6 The relative fluorescence intensity of VAV2 expression.

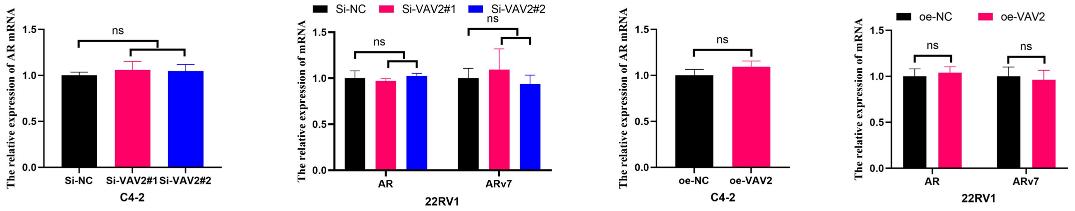

Fig. S7 The levels of AR and ARv7 mRNA after dowregulation and upregulation of VAV2. “ns” is not significant.

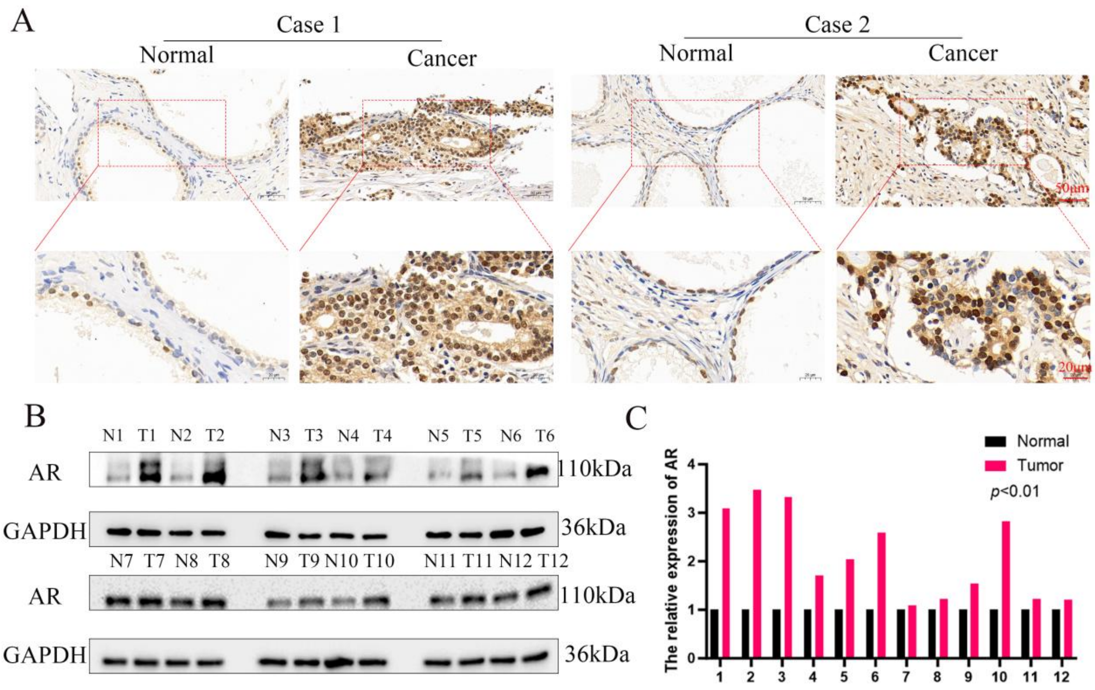

Fig. S8 The expression of AR in patients' tissues. **A** IHC showed the expression of AR in PCa tissues. **B-C** The AR protein level was measured by Western blot.

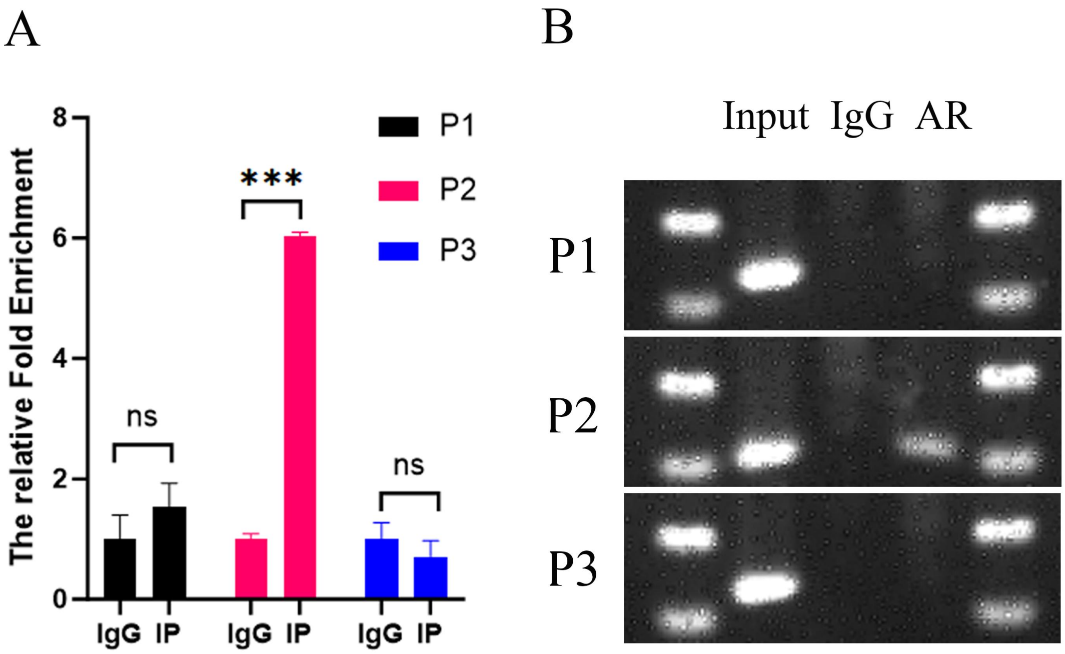

Fig. S9 The results of ChIP in C4-2 cells.
